# The Complex Interplay of Duration of Residence and Region of Birth on Perinatal Outcomes Among Migrant Women in Australia: A Cohort Study

**DOI:** 10.1111/ppe.70205

**Published:** 2026-09-27

**Authors:** Natalie Holowko, Sol P. Juárez, Kirsten R. Palmer, Marian Knight, Fatima El Assaad, Miranda Davies‐Tuck

**Affiliations:** ^1^ The Ritchie Centre Hudson Institute of Medical Research Clayton Australia; ^2^ Department of Obstetrics and Gynaecology Monash University Melbourne Australia; ^3^ Department of Public Health Sciences Stockholm University Stockholm Sweden; ^4^ Centre for Health Equity Studies (CHESS) Stockholm University/Karolinska Institutet Stockholm Sweden; ^5^ Department of Global Public Health Karolinska Institutet Stockholm Sweden; ^6^ National Perinatal Epidemiology Unit, Nuffield Department of Population Health University of Oxford Oxford UK; ^7^ Microbiome Research Centre, St George & Sutherland Clinical Campuses, School of Clinical Medicine, UNSW Medicine & Health UNSW Sydney Sydney Australia; ^8^ The Still Nest Sydney Australia

**Keywords:** antenatal care use, duration of residence, migrant women, perinatal outcomes, region of birth, stillbirth

## Abstract

**Background:**

Although migrants are recognised as heterogeneous, research often emphasises origin over receiving‐context experiences.

**Objectives:**

We investigated maternal risk factors and perinatal outcomes by duration of residence in Australia, stratified by region of birth.

**Methods:**

This population‐based cohort study using Victorian Perinatal Data Collection data included singleton births between 2017–2019 (*N* = 199,935). Exposures were maternal region of birth (United Nations‐grouped countries) and duration of residence (< 2, 2–5, 6–10, > 10 years, [~15% missing]). Outcomes were grouped into preconception health (smoking, body mass index (BMI), pre‐existing conditions, social disadvantage), structural factors (antenatal care, early ultrasound) and pregnancy/perinatal outcomes (gestational diabetes, hypertensive disorders, gestation at birth, stillbirth). Relative risks were estimated using robust Poisson regression, adjusted for maternal age and birth year, stratified by region of birth.

**Results:**

Among the 36.7% of women born outside of Australia, most were from South Asia or South‐East/East Asia, with a mean residence of 10 years. Compared with Australia‐born women, migrant women generally had a lower risk of smoking, pre‐existing hypertension, BMI ≥ 25, preeclampsia and preterm birth—although advantages were less pronounced in women residing longer and varied by region. Some groups had a higher risk of gestational diabetes (Oceania, South Asia, South‐East/East Asia, West Asia) and stillbirth (Oceania, South Asia, Africa), with risks persisting regardless of residence duration. Late initiation of antenatal care was consistently observed among women born in New Zealand, Oceania, Africa, West Asia and Other regions. Sensitivity analyses (i) treating missing duration as a category; (ii) restricting to women migrating ≥ 18 years; and (iii) imputing missing data for duration, BMI and smoking, yielded similar results.

**Conclusions:**

Perinatal outcomes varied by region of birth and duration of residence, indicating the importance of both origin and receiving‐context factors. Improving outcomes requires addressing structural barriers and upstream determinants disproportionately affecting specific migrant groups.

## 1 Background

Ensuring equitable and optimal pregnancy outcomes for migrant populations is a fundamental priority for maternity services worldwide and vital towards building more inclusive and cohesive societies. Risk factors for adverse pregnancy outcomes span multiple domains, including preconception health, nutritional adequacy, psychosocial stress, healthcare access and trust, and the affordability of quality care [1, 2, 3].

Migration is a dynamic process shaped by legal status, policy and the settlement context. The ‘healthy migrant paradox’ suggests that, despite socioeconomic disadvantages, migrants often have better health outcomes than native‐born populations and also fare better than those who choose not to migrate [4]. This may reflect a healthy selection effect, in which migrants are generally healthier and at an age when fewer health issues are present. However, this phenomenon is not universally observed across all migrant backgrounds, contexts, or pregnancy outcomes [5, 6, 7, 8, 9, 10, 11].

The relationship between migration and pregnancy outcomes reveals complex patterns. Beyond individual characteristics, vulnerability is shaped by structural, social and health‐system factors [12], which are dynamic over time and modified by duration of residence. Evidence from high‐income settings shows that initial health advantages observed among new migrants tend to decline with longer time in the host country [13, 14, 15, 16, 17], although this is not universal across outcomes. A Norwegian study [18] found no association between duration and stillbirth, despite some indication of increased risk among nulliparous women who had resided > 5 years (compared to < 2 years). Similarly, a Swedish study [8] found no association with birthweight and that region of birth (Human Development Index defined) had a greater influence than duration of residence. This suggests that inequalities in the country of origin may play a larger role for migrants.

Broad generalisations about increased risks among migrant women and one‐size‐fits‐all approaches overlook heterogeneity between and within migrant groups. As migrants adapt to the host population, they may simultaneously face increased discrimination and health‐affecting challenges, including linguistic and cultural barriers, institutional biases and limited healthcare engagement [19, 20, 21]. Migrant women may experience uncertainty about eligibility for public health insurance (known as Medicare in Australia) [22], lack of qualified interpreters, low trust in healthcare due to past experiences [23] and competing settlement demands [24] such as housing, employment, and childcare. These factors contribute to reduced service use [19] and underreporting of conditions [20], alongside migration‐related trauma [25].

Duration of residence provides a valuable lens to examine this heterogeneity. Variation by duration of residence may reflect changes in diagnosis, health behaviours, and social determinants such as labour market outcomes and education. Regardless of mechanism, duration of residence is a practical tool for monitoring migrant health when individual‐level data are unavailable [26]. Although rarely captured in health registers, it reflects time‐related group differences and provides insight into how prolonged exposure to the host country's healthcare system, environment, and social structures shapes outcomes. To address heterogeneity in pregnancy outcomes among migrants in Australia, we investigated associations between duration of residence and preconception health, structural factors, and pregnancy and birth outcomes, stratified by region of birth.

## 2 Methods

### 2.1 Cohort Selection

This population‐based cohort study used routinely collected data from the Victorian Perinatal Data Collection (VDPC), including all births in Victoria ≥ 20 weeks' gestation (or birth weight ≥ 400 g where gestation is unknown). The VDPC reports > 95% accuracy for 70% of the 111 variables and > 90% accuracy for 86% [27]. Data were reported by Health Services to the Consultative Council on Obstetric and Paediatric Mortality and Morbidity (CCOPMM), with no individual consent required.

The study was drawn from 247,104 births between 2017 and 2019 (Figure 1). We excluded multiple births, data anomalies, congenital anomalies, and terminations. Pregnancies among Aboriginal and/or Torres Strait Islander women were excluded to reduce reference group bias, given distinct historical, social, and systemic determinants of health and perinatal outcomes [28]. After excluding 14,901 women with missing smoking, BMI, or duration of residence data (later included in sensitivity analyses), the final analytical sample comprised 199,235 births.

**FIGURE 1:**
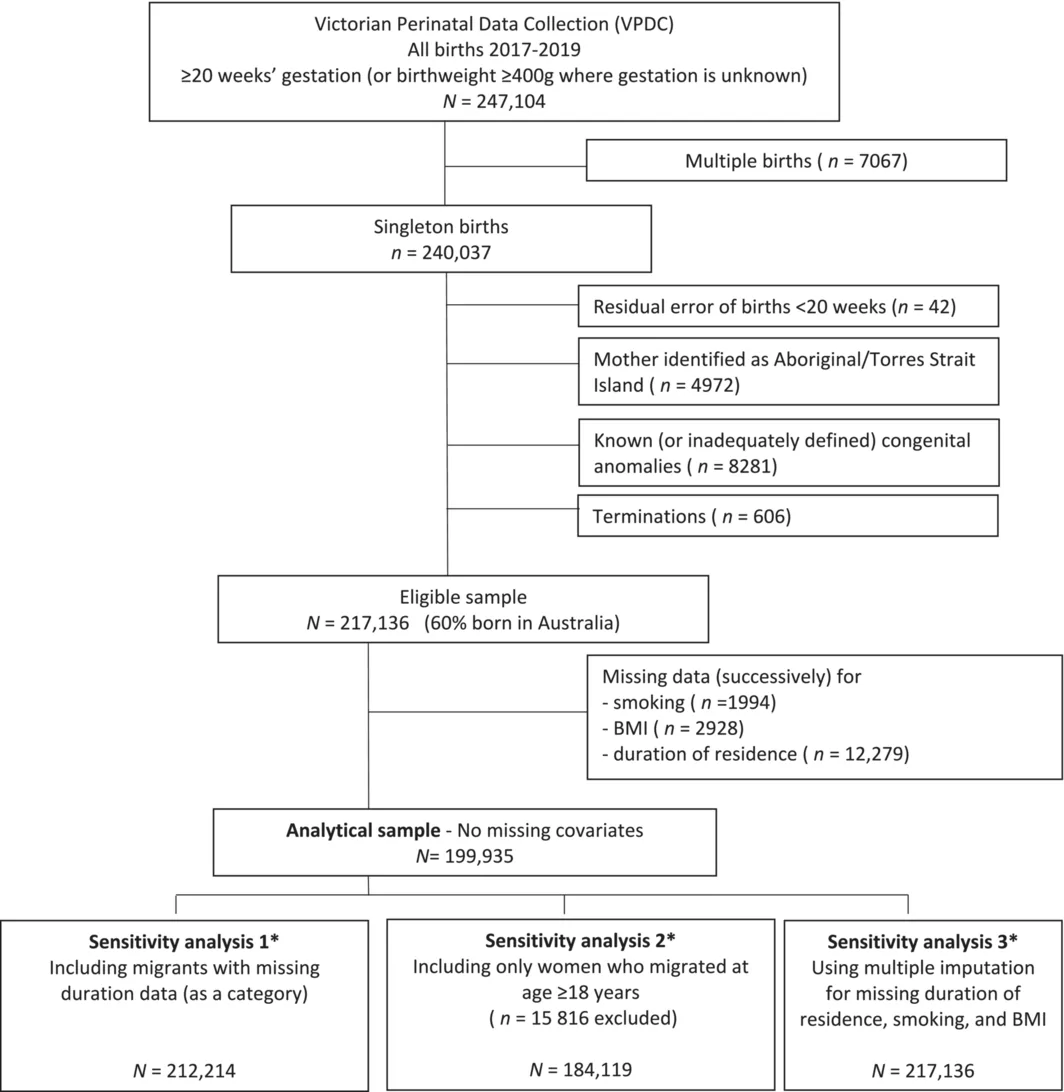
Sample attrition for the study populations used in the main and sensitivity analyses.

### 2.2 Exposures—Markers of Migration

Migrants were women born outside of Australia. We grouped countries into United Nations (UN) defined ‘regions of birth’—Australia, New Zealand, Oceania, Africa, West Asia (including Iran, Iraq, Jordan, Turkey, Yemen, Syria, Cyprus), South Asia (including India, Pakistan, Sri Lanka, Afghanistan, Bangladesh), South‐East and East Asia (including Vietnam, Malaysia, Indonesia, China, Japan), Europe or Other [29]. These groups are simplified for policy relevance, though heterogeneity remains. Non‐indigenous Australia‐born women were the reference group.

Duration of residence was calculated as the difference between the child's birth year and the mother's year of arrival in Australia (reported in the VDPC from 2017 onwards), then categorised (< 2, 2–5, 6–10, > 10 years). Age at arrival in Australia was described.

### 2.3 Outcomes

*Preconception health factors* included social disadvantage, measured using the Indicator for Relative Social Deprivation quintiles (summarising area‐level attributes, including low income, high unemployment, low educational attainment and unskilled occupations), with Q1 indicating the most socially disadvantaged areas [30]; smoking status (non‐smokers [reference] vs. women who had ever smoked [current smokers and those who quit < 20 weeks gestation]); pre‐existing diabetes; pre‐existing hypertension and pre‐existing thyroid conditions. Early‐pregnancy body mass index (BMI) was (i) described using World Health Organisation (WHO) standard categories (< 18.5, 18.5–24.9, 25.0–29.9, and ≥ 30 kg/m^2^); and (ii) modelled as < 25.0 [reference] vs. ≥ 25.0 (corresponding to having an overweight or obese weight status). Women with missing data for smoking (*n* = 1994, 0.92%) and BMI (*n* = 3120, 1.4%) were excluded from the primary analysis (these values were imputed in a sensitivity analysis).

*Structural factors (access to care)* included: gestational age at first antenatal visit (< 12 weeks [reference], 12–17 weeks, ≥ 18 weeks, or none/inadequately defined); antenatal care attendance (no or yes); early ultrasound (< 15 weeks).

*Pregnancy and birth outcomes* modelled were: Gestational diabetes mellitus (GDM); gestational hypertension; preeclampsia; gestational age at birth (preterm (< 37 weeks), term (37–41 weeks), or post‐term (≥ 42 weeks)); stillbirth (fetal death ≥ 20 weeks' gestation or birthweight ≥ 400 g where gestation was unknown).

*Covariates included*: maternal age at birth (< 25, 25–29.9, 30–34.9, 35–35.9, or ≥ 40 years) and birth year of the child (Figure S1).

### 2.4 Statistical Analysis

Preconception health factors, structural factors (access to care), and pregnancy and birth outcomes are presented descriptively and graphically (frequencies and percentages) and stratified by whether the woman was born in Australia and by duration of residence for women born abroad.

Associations between duration of residence and preconception health factors, structural factors and birth outcomes were modelled—overall and stratified by region of birth—using Poisson regression with a log‐link and robust (sandwich) variance estimators. Maternal age and year of birth were conditioned for in all models, with estimated adjusted relative risks (aRR) and 95% confidence intervals (CI) presented. Developed for and commonly applied to network meta‐analysis, we used an adaptation of Vitruvian plots [31] to visualise the strength of the association between duration of residence, preconception health, structural factors (access to care) and pregnancy and birth outcomes overall, including adjusted RRs and 95% CIs. To improve readability, forest plots (with adjusted relative risks (aRRs) plotted on a log‐base 10 scale) were used to visualise models stratified by region of birth.

### 2.5 Sensitivity Analyses

We conducted sensitivity analyses (i) treating missing duration as a category; (ii) restricting to women migrating ≥ 18 years; and (iii) imputing missing data for duration, BMI and smoking (Figure 1).

### 2.6 Missing Data

Duration of residence was missing for 14.8% (*n* = 12,918) of migrant women in the eligible sample, who were excluded in the analytical sample. This missingness was not plausibly Missing At Random and we believe this represents a distinct subgroup of vulnerable women. Therefore, in our first sensitivity analysis, we treated missing duration as a separate category, as imputing exposure variables can introduce bias [32]. Upon request, we also imputed duration of residence in an additional sensitivity analysis, along with two covariates with missing data—smoking (*n* = 1994, 0.92%) and BMI (*n* = 3120, 1.4%). Estimates for the observed duration categories were materially unchanged across approaches, indicating robustness of our findings.

The study was conducted and reported in accordance with STROBE guidelines, using the cohort‐study checklist. Data management and statistical analyses were conducted in SAS 9.4 (SAS Institute Inc., Cary, NC, USA) and R Studio 2024.04.2 (R version 4.4.1 and RStudio Team, Integrated Development for R, Boston, MA, USA, URL http://www.rstudio.com).

### 2.7 Ethics Approval

Ethical approval for this study was obtained from the Monash University Human Research Ethics Committee (#26957, 28 Jan 2021). Access to and analysis of the data were approved by CCOPMM.

## 3 Results

Most of the 199,935 births in Victoria (2017–2019) in the analytical sample were to mothers born outside of Australia, primarily from South Asia (~one‐third) and South‐East/East Asia (~one‐third). Over three‐quarters of migrant women arrived in Australia ≥ 18 years of age, with the mean duration of residence 10 years (IQR 4–12) (Table 1). Approximately 8.5% of migrants had lived in Australia for < 2 years, while the remainder were evenly distributed across the other duration categories.

**TABLE 1:** Maternal and pregnancy characteristics among women who gave birth in Victoria between 2017–2019^a^ by (i) whether they were born in Australia; and (ii) duration of residence (among the 73,314 women who were born abroad) (*N* = 199,935).

	Born in Australia (*N* = 126,621, 63.3%)	Born outside of Australia (*N* = 73,314, 36.7%)	Duration of residence			
			< 2 years (*N* = 6263, 8.5%)	2–5 years (*N* = 21,471, 29.3%)	6–10 years (*N* = 21,582, 29.4%)	> 10 years (*N* = 23,998, 32.7%)
Migration characteristics						
Age at arrival						
< 18 years		15,816 (21.6)	14 (0.2)	283 (1.3)	1303 (6.0)	14,216 (59.2)
≥ 18 years		57,497 (78.4)	6249 (99.8)	21,187 (98.7)	20,279 (94.0)	9782 (40.8)
Missing		1	0 (0.0)	1 (0.0)	0 (0.0)	0 (0.0)
Length of residence (years), Mean (IQR), Median		*N* = 73,314, 10 (4–12), 8	1 (1–1), 1	3 (3–4), 3	8 (7–9), 8	19 (13–25), 17
Region of birth^b^						
New Zealand		3959 (5.4)	174 (2.8)	733 (3.4)	1148 (5.3)	1904 (7.9)
Oceania		1230 (1.7)	101 (1.6)	307 (1.4)	312 (1.4)	510 (2.1)
South‐East/East Asia		22,488 (30.7)	1607 (25.7)	5493 (25.6)	6577 (30.5)	8811 (36.7)
South Asia		24,491 (33.4)	2811 (44.9)	9851 (45.9)	7897 (36.6)	3982 (16.4)
Europe		8177 (11.2)	369 (5.9)	1806 (8.4)	2377 (11.0)	3625 (15.1)
Africa		5804 (7.9)	410 (6.5)	1222 (5.7)	1480 (6.9)	2692 (11.2)
West Asia		3805 (5.2)	568 (9.1)	1140 (5.3)	847 (3.9)	1250 (5.2)
Other		3360 (4.6)	223 (3.6)	919 (4.3)	944 (4.4)	1274 (5.3)
Preconception health and maternal characteristics						
Maternal age (years)						
< 25 years	14,102 (11.1)	5083 (6.9)	1088 (17.4)	2011 (9.4)	880 (4.1)	1104 (4.6)
25–29.9 years	32,242 (25.5)	18,078 (24.7)	2375 (37.9)	7279 (33.9)	4624 (21.4)	3800 (15.8)
30–34.9 years	47,542 (37.5)	29,751 (40.6)	1867 (29.8)	8384 (39.0)	10,016 (46.4)	9484 (39.5)
35–39.9 years	26,582 (21.0)	16,856 (23.0)	788 (12.6)	3194 (14.9)	5213 (24.2)	7661 (31.9)
≥ 40 years	6153 (4.9)	3546 (4.8)	145 (2.3)	603 (2.8)	849 (3.9)	1949 (8.1)
Parity						
0	55,266 (43.6)	31,690 (43.2)	3945 (63.0)	11,095 (51.7)	7923 (36.7)	8727 (36.4)
1	45,834 (36.2)	27,253 (37.2)	1496 (23.9)	7405 (34.5)	9299 (43.1)	9053 (37.7)
2	17,866 (14.1)	8835 (12.1)	509 (8.1)	1968 (9.2)	2831 (13.1)	3527 (14.7)
≥ 3	7652 (6.0)	5533 (7.5)	312 (5.0)	1003 (4.7)	1529 (7.1)	2689 (11.2)
Missing		3 (0.0)	1 (0.0)	0 (0.0)	0 (0.0)	2 (0.0)
Smoking status at 20 weeks gestation						
Non‐smoker	107,882 (85.2)	69,874 (95.3)	6062 (96.8)	20,780 (96.8)	20,732 (96.1)	22,300 (92.9)
Quit smoking < 20 weeks	2406 (1.9)	596 (0.8)	35 (0.6)	139 (0.6)	142 (0.7)	280 (1.2)
Smoker	16,333 (12.9)	2844 (3.9)	166 (2.7)	558 (2.6)	708 (3.3)	1418 (5.9)
Body mass index (kg/m^2^)						
< 18.5	2668 (2.1)	2846 (3.9)	349 (5.6)	901 (4.2)	793 (3.7)	803 (3.3)
18.5–24.9	57,316 (45.3)	39,014 (53.2)	3475 (55.5)	11,759 (54.8)	11,370 (52.7)	12,410 (51.7)
25.0–29.9	35,479 (28.0)	20,455 (27.9)	1713 (27.4)	5984 (27.9)	6285 (29.1)	6473 (27.0)
≥ 30	31,158 (24.6)	10,999 (15.0)	726 (11.6)	2827 (13.2)	3134 (14.5)	4312 (18.0)
Missing						
Pre‐existing diabetes mellitus	1023 (0.8)	742 (1.0)	33 (0.5)	193 (0.9)	243 (1.1)	273 (1.1)
Pre‐existing hypertension	1662 (1.3)	616 (0.8)	34 (0.5)	128 (0.6)	172 (0.8)	282 (1.2)
Thyroid conditions	6300 (5.0)	6257 (8.5)	462 (7.4)	2069 (9.6)	2082 (9.6)	1644 (6.9)
Access to care and social disadvantage						
Early ultrasound (< 15 weeks)	88,359 (69.8)	46,924 (64.0)	3772 (60.2)	14,027 (65.3)	14,099 (65.3)	15,026 (62.6)
Antenatal Care attendance	126,018 (99.5)	73,069 (99.7)	6230 (99.5)	21,401 (99.7)	21,534 (99.8)	23,904 (99.6)
Gestational age at first antenatal visit						
< 12 weeks	81,596 (64.4)	40,058 (54.6)	2864 (45.7)	11,159 (52.0)	11,952 (55.4)	14,083 (58.7)
12–17 weeks	30,581 (24.2)	21,567 (29.4)	1575 (25.1)	6665 (31.0)	6537 (30.3)	6790 (28.3)
≥ 18 weeks	13,844 (10.9)	11,444 (15.6)	1791 (28.6)	3577 (16.7)	3045 (14.1)	3031 (12.6)
None/inadequately defined	600 (0.5)	245 (0.3)	33 (0.5)	70 (0.3)	48 (0.2)	94 (0.4)
IRSD (Indicator of relative social deprivation) *N* = 198,532						
1	21,574 (17.1)	17,834 (24.6)	1996 (32.5)	6080 (28.7)	4913 (23.0)	4845 (20.3)
2	25,598 (20.3)	14,480 (19.9)	1332 (21.7)	4484 (21.1)	4303 (20.1)	4361 (18.3)
3	26,355 (20.9)	13,931 (19.2)	1123 (18.3)	4033 (19.0)	4222 (19.7)	4553 (19.1)
4	25,506 (20.3)	13,983 (19.3)	971 (15.8)	3682 (17.4)	4270 (20.0)	5060 (21.2)
5	26,902 (21.4)	12,369 (17.0)	729 (11.9)	2938 (13.8)	3679 (17.2)	5023 (21.1)
Pregnancy and birth outcomes						
Gestational diabetes mellitus	12,322 (9.7)	14,737 (20.1)	1069 (17.1)	4387 (20.4)	4545 (21.1)	4736 (19.7)
Gestational hypertension	3838 (3.0)	1141 (1.6)	78 (1.2)	273 (1.3)	325 (1.5)	465 (1.9)
Preeclampsia	2868 (2.3)	1185 (1.6)	111 (1.8)	335 (1.6)	306 (1.4)	433 (1.8)
Gestation of birth						
Preterm (< 37 weeks)	7983 (6.3)	4087 (5.6)	330 (5.3)	1177 (5.5)	1229 (5.7)	1351 (5.6)
Term (37–41 weeks)	118,256 (93.4)	69,035 (94.2)	5923 (94.6)	20,244 (94.3)	20,289 (94.0)	22,579 (94.1)
Post‐term (≥ 42 weeks)	382 (0.3)	192 (0.3)	10 (0.2)	50 (0.2)	64 (0.3)	68 (0.3)
Stillbirth	426 (0.3)	284 (0.4)	28 (0.4)	68 (0.3)	87 (0.4)	101 (0.4)

^a^ Excluding births: before 20 weeks' gestation; to indigenous mothers; with congenital anomalies (or inadequately defined); terminations of pregnancy.

^b^ Region of birth categorised according to UN groupings: Australia, New Zealand, Oceania, Africa, West Asia (including Iran, Iraq, Jordan, Turkey, Yemen, Syria, Cyprus), South Asia (including India, Pakistan, Sri Lanka, Afghanistan, Bangladesh), South‐East and East Asia (including Vietnam, Malaysia, Indonesia, China, Japan), Europe or Other.

The composition of migrants changed with the duration of residence. While the highest percentage of women residing > 10 years were born in South‐East/East Asia, in more recent years an increasing percentage of women were born in South Asia and West Asia (Table 1).

### 3.1 Migration, Duration of Residence and Preconception Health

Overall, compared with women born in Australia, fewer migrant women had pre‐existing hypertension (Table 1). While a higher percentage of migrant women had a BMI within the recommended range (53.2% vs. 45.3%), they were also more likely to have pre‐existing diabetes (1.0% vs. 0.8%) or a thyroid condition (8.5% vs. 5.0%). Migrant women were less likely to smoke during pregnancy, have pre‐existing hypertension and have a BMI ≥ 25, and this was slightly stronger among recently arrived migrants (Figure 2a). In contrast, the risk of pre‐existing diabetes was higher among women residing ≥ 2 years, and there was a persistent increased relative risk of pre‐existing thyroid conditions that was highest among migrant women residing 2–10 years (Figure 2a).

**FIGURE 2:**
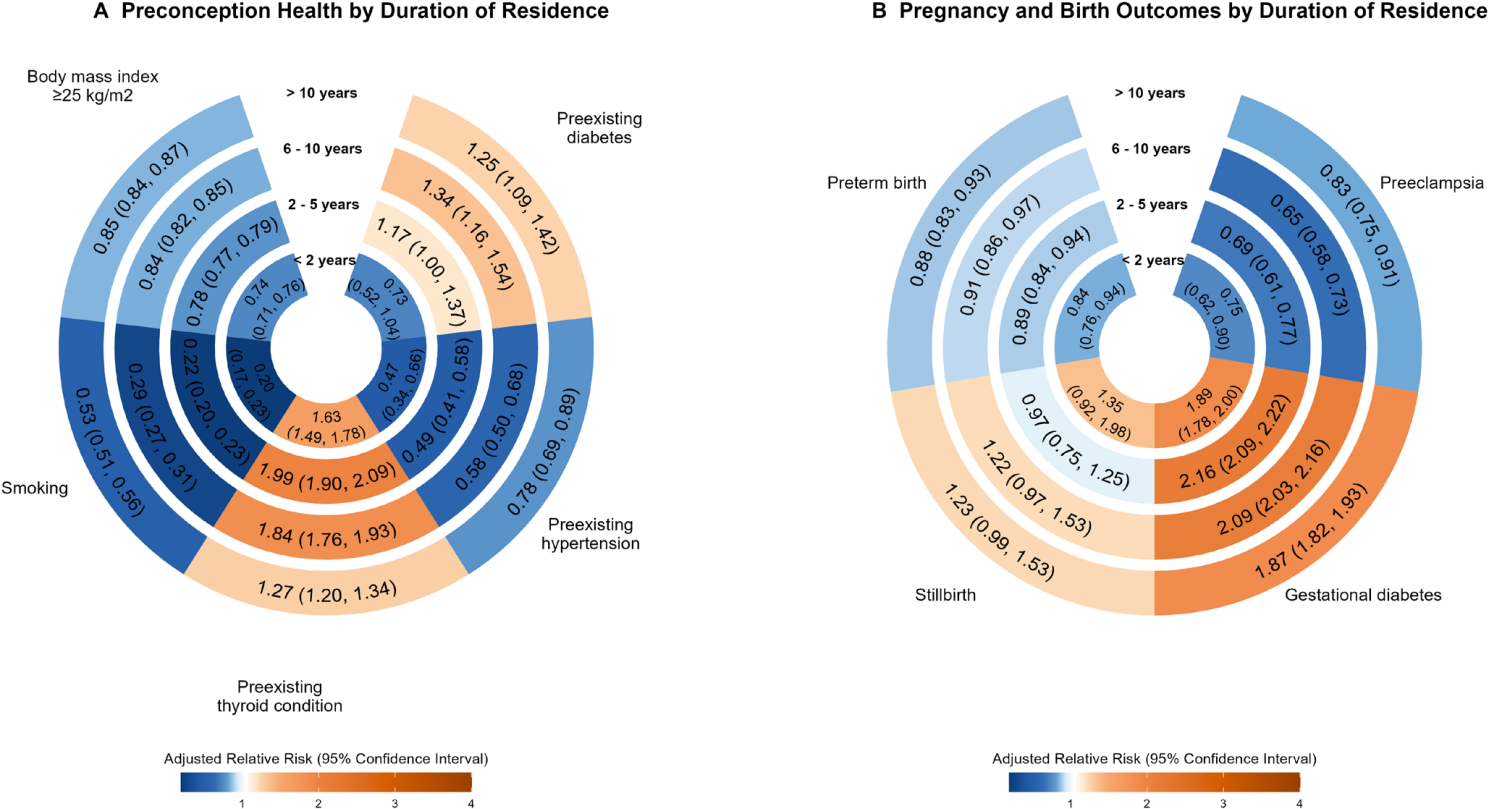
(A and B) Plots showing the overall adjusted relative risk of preconception health and pregnancy/birth outcomes by duration of residence, compared to Australia‐born women. Each segment represents a specific preconception health indicator (A) or birth outcome (B). Within each segment, the four concentric bands correspond to duration of residence, from < 2 years (innermost band) to > 10 years (outermost band). The colour reflects the direction and magnitude of the association compared with women born in Australia: Blue indicates lower risk and orange indicates higher risk, with darker shades representing stronger associations than the reference group (Australia‐born women).

Overall, more women born abroad experienced the greatest social disadvantage (IRSD 1 and 2, 44.5% vs. 37.4% of Australia‐born women), with women residing < 2 years being most disadvantaged. The disparity in social deprivation between migrant and Australia‐born women appeared less pronounced among those residing > 10 years (Table 1).

### 3.2 Migration, Duration of Residence and Preconception Health, Stratified by Region of Birth

While migrant women had a lower risk of smoking during pregnancy, compared to Australia‐born women, a higher risk was observed among women born in New Zealand with shorter and longer duration of residence (Figure 3, Table S1).

**FIGURE 3:**
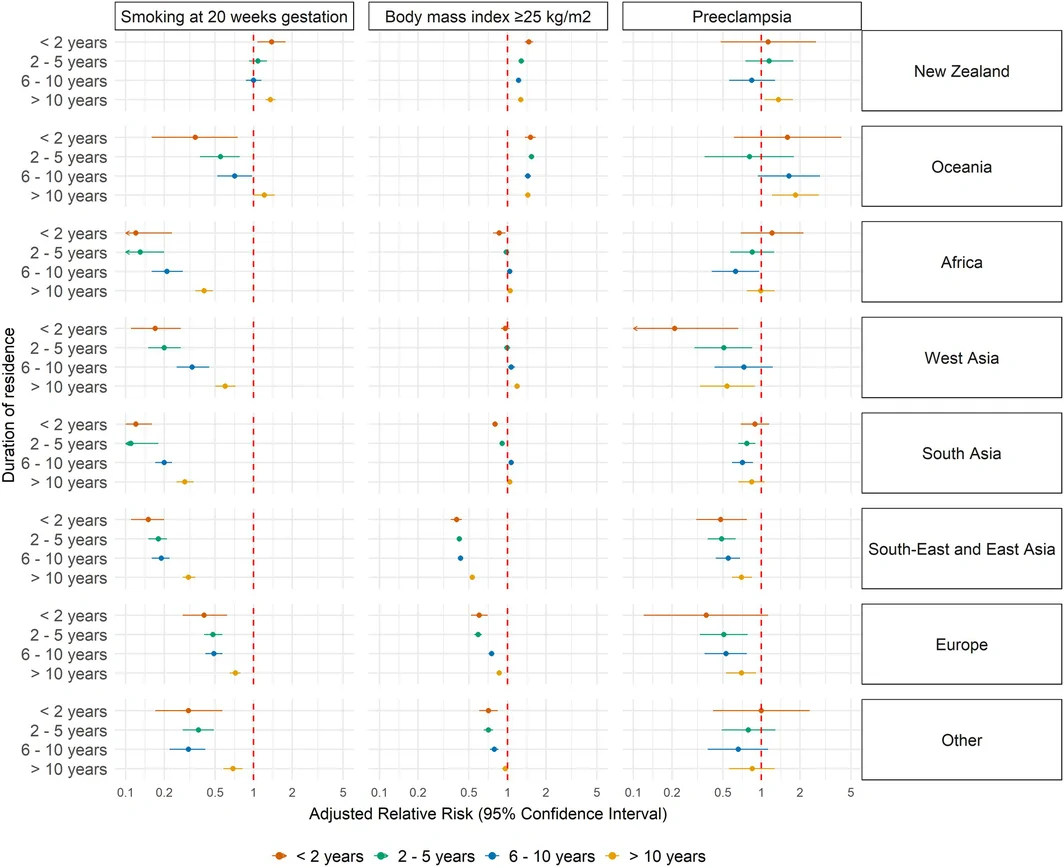
Forest plot showing the adjusted relative risk (and 95% confidence intervals) of smoking at 20 weeks gestation, Body mass index ≥ 25 kg/m^2^ and preeclampsia by duration of residence, stratified by region of birth.

Similarly, migrant women had a lower relative risk of pre‐existing hypertension, with minor differences by duration of residence. Women born in South‐East/East Asia, Europe and South Asia stand out as having generally had lower risk, with the exception of South Asia‐born women who had resided in Australia > 10 years (aRR 1.23, 95% CI 0.97, 1.55) (Figure 4, Table S1).

**FIGURE 4:**
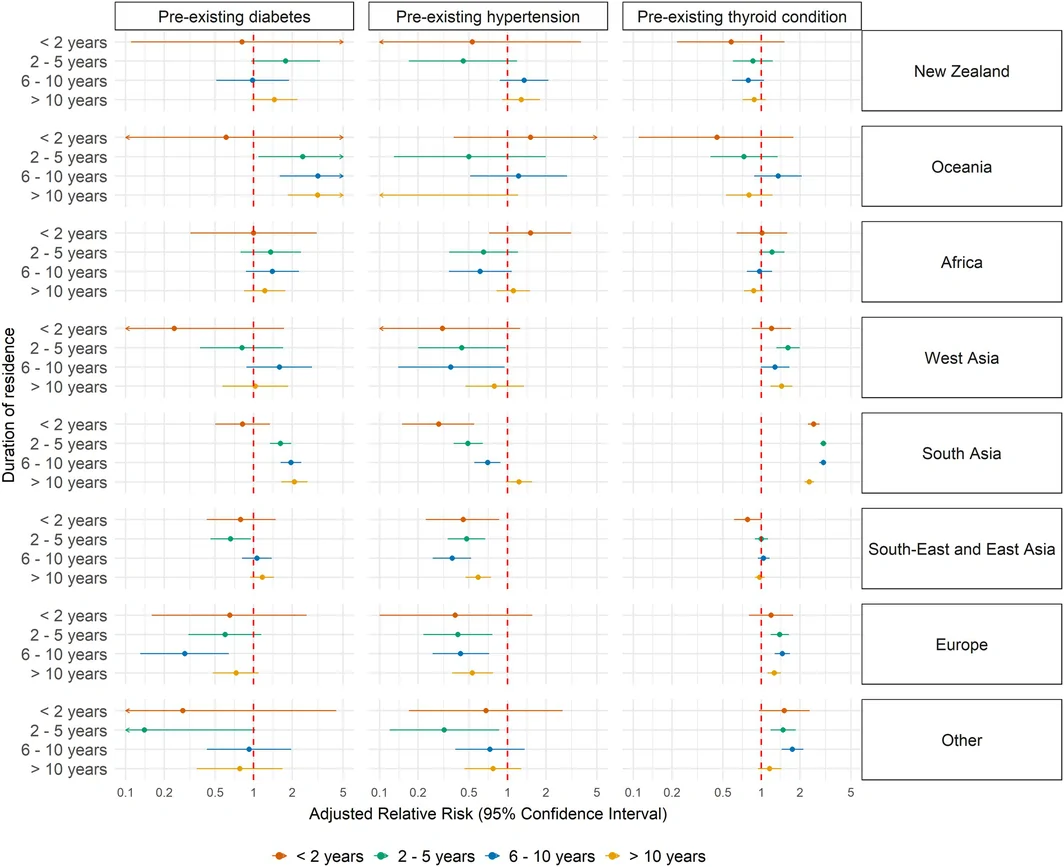
Forest plot showing the adjusted relative risk (and 95% confidence intervals) of pre‐existing diabetes, pre‐existing hypertension and pre‐existing thyroid conditions by duration of residence, stratified by region of birth.

Regardless of duration of residence, women born in New Zealand and Oceania consistently had an increased risk of having a BMI ≥ 25.0 (Figure 3, Table S1) and there was some indication of increased risk among women residing in Australia for > 5 years who were born in Africa, West Asia or South Asia. Duration of residence ≥ 2 years was associated with higher risk of pre‐existing diabetes among women born in Oceania and South Asia residing in Australia > 2 years (Figure 4, Table S1).

### 3.3 Migration, Duration of Residence and Access to Care

While almost all women attended antenatal care, fewer migrants had their first antenatal visit < 12 weeks of gestation (54.6% vs. 64.4% of women born in Australia) (Table 1). Increased risk of late antenatal care initiation (≥ 18 weeks' gestation) attenuated the longer a person had lived in Australia (Table S2).

### 3.4 Migration, Duration of Residence and Access to Care, Stratified by Region of Birth

Women born in New Zealand, West Asia and Africa consistently had the highest relative risk (2 to 3 times higher) of late antenatal care initiation (Table S2), which was highest among women who had resided < 2 years. This pattern was consistent across regions, including for women born in Oceania, who also had a high risk of engaging in antenatal care at ≥ 18 weeks' gestation (2 to 4 times the risk) and women born in South‐East/East Asia (1.4 to 2.7 times the risk).

### 3.5 Migration, Duration of Residence, Pregnancy and Birth Outcomes

Overall, compared to Australia‐born women, migrant women were less likely to be diagnosed with gestational hypertension, preeclampsia and preterm birth and more likely to experience gestational diabetes (Table 1, Figure 2a,b). No differences were found in the risk of stillbirth.

The association between preeclampsia, preterm birth, gestational diabetes and stillbirth did not differ substantially by duration (Figure 2b, Table S3). In a sensitivity analysis, the risk of stillbirth was increased for women with missing duration of residence (almost twice the risk) (Figure 2b, Table S4).

### 3.6 Migration, Duration of Residence, Pregnancy and Birth Outcomes, Stratified by Region of Birth

The association between duration of residence and pregnancy outcomes differed by maternal region of birth (Figure 3, Figure 5, Table S3). Increased risk of gestational diabetes was consistently found among women born in Oceania (aRRs ranged between 2.28 and 2.56), South Asia (aRRs ranged between 2.33 and 2.96), West Asia (aRRs ranged between 1.57 and 1.98), and South‐East/East Asia (aRRs ranged between 1.80 and 2.05) (Figure 5, Table S3). Among women born in Africa, a slightly increased risk was observed for those who had resided ≥ 2 years (aRRs ranged from 1.33 to 1.58).

**FIGURE 5:**
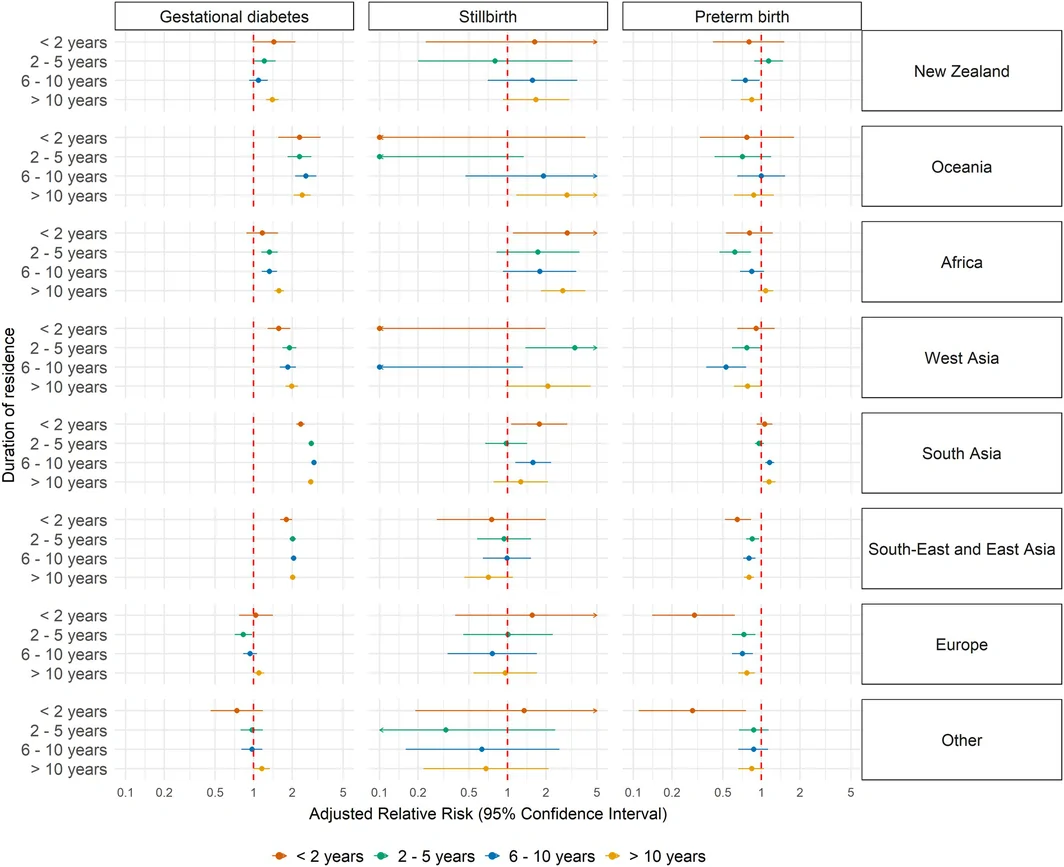
Forest plot showing the adjusted relative risk (and 95% confidence intervals) of gestational diabetes, stillbirth and preterm birth by duration of residence, stratified by region of birth.

Regardless of duration of residence, a consistently reduced risk of preeclampsia was observed among women born in South‐East/East Asia (RRs ranged from 0.48 to 0.70) (Figure 3, Table S3). Reduced risk of preeclampsia was also found among West Asia‐born women (aRRs ranged from 0.21 to 0.54) and women born in Europe residing > 2 years (aRRs ranged from 0.51 to 0.70). South Asia‐born women residing 2–10 years had reduced risk of preeclampsia (aRRs ranged between 0.71 and 0.77).

Overall, migrant women had a lower risk of preterm birth (Figure 2b). When considering variation by region and duration of residence, this was specific to women born in South‐East/East Asia (aRRs ranged from 0.65 to 0.85) and Europe (aRRs ranged from 0.30 to 0.77) (Figure 5, Table S3). Among West Asia‐born women, lower risk of preterm birth was observed in women residing ≥ 2 years (2–5 years: aRR 0.77, 95% CI 0.59, 0.99, > 5–10 years: aRR 0.53, 95% CI 0.37, 0.76).

Regardless of duration, increased risk of stillbirth was found in women born in Africa (< 2 years: aRR 2.93, 95% CI 1.10, 7.79 and > 10 years: aRR 2.71, 95% CI 1.82, 4.05) (Figure 5, Table S3). Increased risk of stillbirth was also found in South Asia‐born women residing < 2 years (aRR 1.77, 95% CI 1.07, 2.93) or 6–10 years (aRR 1.58, 95% CI 1.15, 2.19) and Oceania‐born women residing > 10 years (aRR 2.89, 95% CI 1.20, 6.95).

For all outcomes, similar associations were found in sensitivity analyses (Figure 1) (i) treating missing duration as a category (Tables S4–S6); (ii) restricting to women migrating ≥ 18 years (Tables S7–S9); and (iii) imputing missing data for duration, BMI and smoking (Tables S10–12). This supports the robustness of our main findings and suggests our estimates likely underestimate the association.

## 4 Comment

### 4.1 Principal Findings

This population‐based cohort of 199,935 women giving birth in Victoria (2017–2019) shows that preconception health, antenatal care use and pregnancy/birth outcomes differ between migrant and Australia‐born women, varying by duration of residence and region of birth. Migrant women generally had more favourable preconception health profiles, though these advantages were less pronounced among women residing in Australia longer and differed across regions of birth. Antenatal care attendance improved for most migrant groups, while higher risks of pre‐existing diabetes, gestational diabetes and stillbirth were more common among women residing longer, with region‐specific patterns. These findings highlight substantial heterogeneity across migrant populations and the complex interplay between migration and duration of residence. Although not causal, the results underscore important time‐related and contextual influences on maternal and perinatal health.

### 4.2 Strengths of the Study

Our study used validated, population‐based data, including all pregnancies in Victoria between 2017 and 2019 and is strengthened by using information on both country of birth (classified into regions) and duration of residence.

### 4.3 Limitations of the Data

A well‐recognised limitation is the composition of the comparison group. We used Australia‐born women as the reference group, acknowledging this does not fully reflect the diverse ethnic identities or racialised experiences influencing healthcare access and outcomes within our heterogeneous host population. The reference population may include minority‐ethnic women experiencing systemic disadvantage/discrimination and adverse health outcomes, introducing exposure misclassification and likely underestimating specific vulnerabilities. Lack of ethnicity data further contributes to exposure misclassification. Migrant group composition also varied by duration of residence, with recent arrivals increasingly originating from South and West Asia. Using duration of residence as a proxy for time‐related variation assumes uniform experiences within regions and simplifies complex migration and settlement processes. The two‐year window of available duration of residence data limited our observation of individual change, although sibling‐based studies suggest duration of residence is a reasonable indicator of individual changes over time [8]. Although age at arrival was not included, sensitivity analysis excluding women who migrated < 18 years indicate our estimates are robust and likely conservative. Small numbers in some region‐stratified groups warrant confirmation in larger studies. Women with missing duration of residence data (15%) may represent administrative invisibility linked to recent arrival or visa insecurity; when included as a category, a higher proportion of these women first attended antenatal care at > 12 weeks and had preeclampsia, a stillbirth, or preterm birth. Additional sensitivity analyses excluding women who migrated < 18 years of age and using multiple imputation for missing duration of residence, BMI, and smoking produced similar estimates to the main analyses.

### 4.4 Interpretation

Our findings support growing evidence that migration‐related factors dynamically shape maternal and perinatal health. Regardless of duration of residence, women born in South‐East/East Asia or West Asia had lower preeclampsia risk, and women born in South‐East/East Asia or Europe had reduced preterm birth risk. Although diminishing advantages among migrant women have been reported [13, 33], our results suggest acculturation alone cannot explain changing risks. For example, increased risk of pre‐existing diabetes among women born in Oceania and South Asia after ≥ 2 years in Australia may reflect delayed diagnosis and care access, alongside growing metabolic risk, indicating missed opportunities for early intervention. Systematically higher gestational diabetes risk among women born in West Asia, South‐East/East Asia, South Asia, Oceania and Africa (> 2 years residence) highlights the need to investigate structural barriers to screening and management and to strengthen early preconception engagement.

Disparities were not uniform across regions or outcomes. Antenatal care engagement was generally better among women who had lived in Australia longer, for example women born in South Asia, South‐East/East Asia, or Europe who had resided > 10 years and showed no difference to Australia‐born women. In contrast, persistent late antenatal care initiation was observed among women born in Africa, Oceania, West Asia and New Zealand. Although improved attendance might be expected to reduce adverse outcomes through earlier detection, this was not consistent across outcomes. While numbers were small for some regions, persistent inequities among specific regions may reflect broader systemic factors—visa status, health insurance eligibility, or racialised maternity care—rather than biological or cultural differences.

Our nuanced findings highlight that neither duration of residence nor region of birth alone adequately explains maternal health outcomes; their combination reveals vulnerable points in the migration experience where risks emerge. Region of birth, often used as a proxy for cultural background, does not capture the diversity within migrant groups, who differ in language, religion, migration pathways, socioeconomic position and access to care. Experiences are also shaped by emotional and cultural factors; for stillbirth, this may include stigma, silence, and limited bereavement care. More granular intersectional measures—such as English proficiency, health literacy, religious affiliation, experiences of racism and settlement context—are needed to inform responsive care.

## 5 Conclusions

Migrant health exists on a complex trajectory shaped by region of birth and time since migration, which together influence preconception health, antenatal care use and pregnancy/birth outcomes. Without targeted action, migrant‐specific structural barriers will continue to drive persistent inequities. Delivering woman‐centred, culturally responsive maternity care requires system‐level solutions that address structural determinants rather than relying on cultural awareness alone or reinforcing simplified narratives of migrant health. Migrant women are not passive recipients of care, but active agents navigating systems not designed for them. From pre‐conception to bereavement, responsive care must reflect women's lived maternity and migration experiences, ensuring culturally safe, interpreter‐enabled services designed with migrant communities to meet their needs.

## Author Contributions

Conceptualisation and planning: Miranda Davies‐Tuck and Natalie Holowko. Data curation: Natalie Holowko. Formal analysis: Natalie Holowko, Miranda Davies‐Tuck and Sol P. Juárez; Funding acquisition: Miranda Davies‐Tuck. Writing – original draft: Natalie Holowko. Writing – review and editing: Natalie Holowko, Miranda Davies‐Tuck, Sol P. Juárez, Kirsten R. Palmer, Marian Knight, Fatima El Assaad. All authors read and approved the final manuscript.

## Funding

M.D.‐T. received support from a National Health and Medical Research Council (#APP2023288) Ideas Grant. M.K. is an NIHR Senior Investigator (NIHR303806). The views expressed in this publication are those of the author(s) and not necessarily those of CCOPMM, the NHS, NIHR or the Department of Health and Social Care.

## Conflicts of Interest

The authors declare no conflicts of interest.

## Supporting information


**Table S1:** The association between duration of residence and preconception health factors (ref = no) stratified by maternal region of birth, compared with women born in Australia. Adjusted relative risks (aRR) and 95% confidence intervals (CI) from robust Poisson^a^ regression models (adjusted for maternal age and year of birth of the child) (*N* = 199,935).
**Table S2:** The association between duration of residence and gestational week of first antenatal care attendance, overall and stratified by maternal region of birth, compared with women born in Australia and first attending antenatal care < 12 weeks' gestation. Adjusted relative risks (aRR) and 95% confidence intervals (CI) from robust Poisson^a^ regression models (adjusted for maternal age and year of birth of the child) (*N* = 199,935).
**Table S3:** The association between duration of residence and pregnancy and birth outcomes (ref = no) stratified by maternal region of birth, compared with women born in Australia. Adjusted relative risks (aRR) and 95% confidence intervals (CI) from robust Poisson^a^ regression models (adjusted for maternal age and year of birth of the child) (*N* = 199,935).
**Table S4:** Sensitivity Analysis 1: The association between duration of residence and preconception health factors (ref = no), stratified by maternal region of birth, compared with women born in Australia. Adjusted relative risks (aRR) and 95% confidence intervals (CI) from robust Poisson^a^ regression models (adjusted for maternal age and year of birth of the child). Results from models in a sensitivity analysis including women with missing duration as a category, but excluding observations missing information on smoking and BMI (*N* = 212,214).
**Table S5:** Sensitivity Analysis 1: The association between duration of residence and gestational week of first antenatal care attendance, overall and stratified by maternal region of birth, compared with women born in Australia and first attending antenatal care < 12 weeks' gestation. Adjusted relative risks (aRR) and 95% confidence intervals (CI) from robust Poisson^a^ regression models (adjusted for maternal age and year of birth of the child). Results from models in a sensitivity analysis including women with missing duration as a category, excluding observations missing information on smoking and BMI (*N* = 212,214).
**Table S6:** Sensitivity Analysis 1: The association between duration of residence and pregnancy and birth outcomes (ref = no), overall and stratified by maternal region of birth, compared with women born in Australia. Relative risks (aRR) and 95% confidence intervals (CI) from robust Poisson^a^ regression models (adjusted for maternal age and year of birth of the child). Results from models in a sensitivity analysis including women with missing duration as a category, excluding observations missing information on smoking and BMI (*N* = 212,214).
**Table S7:** Sensitivity Analysis 2: The association between duration of residence and preconception health factors (ref = no), stratified by maternal region of birth, compared with women born in Australia. Adjusted relative risks (aRR) and 95% confidence intervals (CI) from robust Poisson^a^ regression models (adjusted for maternal age and year of birth of the child) in a sensitivity analysis including only women who migrated at ≥ 18 years of age (*N* = 184,119).
**Table S8:** Sensitivity Analysis 2: The association between duration of residence and gestational week of first antenatal care attendance, overall and stratified by maternal region of birth, compared with women born in Australia and first attending antenatal care < 12 weeks' gestation. Adjusted relative risks (aRR) and 95% confidence intervals (CI) from robust Poisson^a^ regression models (adjusted for maternal age and year of birth of the child)) in a sensitivity analysis including only women who migrated at ≥ 18 years of age (*N* = 184,119).
**Table S9:** Sensitivity Analysis 2: The association between duration of residence and pregnancy and birth outcomes, overall and stratified by maternal region of birth (ref = no), compared with women born in Australia. Adjusted relative risks (aRR) and 95% confidence intervals (CI) from robust Poisson^a^ regression models (adjusted for maternal age and year of birth of the child) in a sensitivity analysis including only women who migrated at ≥ 18 years of age (*N* = 184,119).
**Table S10:** Sensitivity Analysis 3: he association between duration of residence and preconception health factors (ref = no), stratified by maternal region of birth, compared with women born in Australia. Adjusted relative risks (aRR) and 95% confidence intervals (CI) from robust Poisson^a^ regression models (adjusted for maternal age and year of birth of the child) from a sensitivity analysis using imputed data (50 imputations) for missing duration of residence, smoking and OWOB (*N* = 217,136).
**Table S11:** Sensitivity Analysis 3: The association between duration of residence and gestational week of first antenatal care attendance, overall and stratified by maternal region of birth, compared with women born in Australia and first attending antenatal care < 12 weeks' gestation. Adjusted relative risks (aRR) and 95% confidence intervals (CI) from robust Poisson^a^ regression models (adjusted for maternal age and year of birth of the child) from a sensitivity analysis using imputed data (50 imputations) for missing duration of residence, smoking and OWOB (*N* = 217,136).
**Table S12:** Sensitivity Analysis 3: The association between duration of residence and pregnancy and birth outcomes, overall and stratified by maternal region of birth, compared with women born in Australia. Adjusted relative risks (aRR) and 95% confidence intervals (CI) from robust Poisson^a^ regression models (adjusted for maternal age and year of birth of the child) from a sensitivity analysis using imputed data (50 imputations) for missing duration of residence, smoking and OWOB (*N* = 217,136).
**Figure S1:** Directed acyclic graph (DAG) showing the association between duration of residence stratified by region of birth and the three groups of outcomes: preconception health, structural factors and pregnancy/perinatal outcomes.

## Data Availability

The data used for this study are available from the Clinical Councils Unit at the Victorian Government Department of Health and Human Services. Contact details for the owner of the data are: Email: clinical.councils@dhhs.vic.gov.au or Email: consultative.councils@safercarevic.gov.au.
