# Supplementary material for: The Complex Interplay of Duration of Residence and Region of Birth on Perinatal Outcomes Among Migrant Women in Australia: A Cohort Study
Source: Paediatr Perinat Epidemiol. 2026 Sep 27;40(8):918–31. doi: 10.1111/ppe.70205 (PMC13763149; doi:10.1111/ppe.70205)
Supplement: Supplementary file 1 [file PPE-40-918-s001.docx]

**Table S1.** The association between duration of residence and preconception health factors (ref = no) stratified by maternal region of birth, compared with women born in Australia. Adjusted relative risks (aRR) and 95% confidence intervals (CI) from robust Poisson^a^ regression models (adjusted for maternal age and year of birth of the child) (*N* = 199,935).

|  | **Pre-existing diabetes** | | | **Pre-existing hypertension** | | **Pre-existing thyroid condition** | | **Smoking^b^** | | **Early-pregnancy weight status BMI ≥25 kg/m^2^** | |
| --- | --- | --- | --- | --- | --- | --- | --- | --- | --- | --- | --- |
|  | **aRR** | **95% CI** | | **aRR** | **95% CI** | **aRR** | **95% CI** | **aRR** | **95% CI** | **aRR** | **95% CI** |
| ***Duration of residence stratified by region of birth*** | | | | |  |  |  |  |  |  |  |
| **Australia born** | 1.00 (reference) |  | | 1.00 (reference) |  | 1.00 (reference) |  | 1.00 (reference) |  | 1.00 (reference) |  |
|  |  |  | |  |  |  |  |  |  |  |  |
| **New Zealand** | 1.36 | (1.01, 1.85) | | 1.13 | (0.87, 1.47) | 0.84 | (0.72, 0.98) | 1.21 | (1.13, 1.29) | 1.27 | (1.24, 1.30) |
| *<2 years* | 0.81 | (0.11, 5.72) | | 0.53 | (0.07, 3.75) | 0.58 | (0.22, 1.52) | 1.38 | (1.07, 1.78) | 1.47 | (1.37, 1.59) |
| *2-5 years* | 1.78 | (0.96, 3.30) | | 0.45 | (0.17, 1.19) | 0.86 | (0.60, 1.23) | 1.08 | (0.92, 1.27) | 1.28 | (1.22, 1.35) |
| *6-10 years* | 0.98 | (0.51, 1.89) | | 1.35 | (0.87, 2.09) | 0.79 | (0.59, 1.05) | 1.00 | (0.87, 1.15) | 1.22 | (1.16, 1.27) |
| *>10 years* | 1.45 | (0.96, 2.19) | | 1.28 | (0.91, 1.80) | 0.88 | (0.71, 1.08) | 1.35 | (1.24, 1.48) | 1.27 | (1.23, 1.31) |
|  |  |  | |  |  |  |  |  |  |  |  |
| **Oceania** | 2.78 | (1.91, 4.06) | | 0.57 | (0.30, 1.09) | 0.90 | (0.69, 1.17) | 0.83 | (0.72, 0.96) | 1.48 | (1.43, 1.52) |
| *<2 years* | Non-estimable^a^ | | | 1.64^a^ | (0.42, 6.51) | 0.45 | (0.11, 1.78) | 0.35 | (0.16, 0.75) | 1.51 | (1.37, 1.66) |
| *2-5 years* | 2.56\|* | (1.16, 6.57) | | 0.54^a^ | (0.14, 2.13) | 0.73 | (0.40, 1.34) | 0.55 | (0.38, 0.78) | 1.54 | (1.46, 1.62) |
| *6-10 years* | 3.24* | (1.63, 6.44) | | 1.25^a^ | (0.52, 2.97) | 1.35 | (0.88, 2.06) | 0.71 | (0.52, 0.97) | 1.44 | (1.36, 1.53) |
| *>10 years* | 3.07* | (1.79, 5.27) | | Non-estimable^a^ | | 0.80 | (0.53, 1.22) | 1.21 | (1.00, 1.46) | 1.44 | (1.37, 1.51) |
|  |  |  | |  |  |  |  |  |  |  |  |
|  |  |  | |  |  |  |  |  |  |  |  |
| **Africa** | 1.28 | (0.99, 1.65) | | 0.91 | (0.72, 1.16) | 0.98 | (0.87, 1.10) | 0.28 | (0.24, 0.31) | 1.02 | (0.99, 1.04) |
| *<2 years* | 1.00 | (0.32, 3.11) | | 1.51 | (0.72, 3.14) | 1.01 | (0.64, 1.59) | 0.12 | (0.06, 0.23) | 0.86 | (0.77, 0.96) |
| *2-5 years* | 1.36 | (0.79, 2.34) | | 0.65 | (0.35, 1.21) | 1.21 | (0.96, 1.52) | 0.13 | (0.09, 0.20) | 0.98 | (0.93, 1.03) |
| *6-10 years* | 1.40 | (0.87, 2.26) | | 0.61 | (0.35, 1.08) | 0.97 | (0.77, 1.21) | 0.21 | (0.16, 0.28) | 1.04 | (1.00, 1.09) |
| *>10 years* | 1.22 | (0.84, 1.77) | | 1.11 | (0.82, 1.50) | 0.87 | (0.73, 1.04) | 0.41 | (0.35, 0.48) | 1.05 | (1.01, 1.08) |
|  |  |  | |  |  |  |  |  |  |  |  |
| **West Asia** | 1.01 | (0.70, 1.44) | | 0.54 | (0.37, 0.79) | 1.42 | (1.26, 1.60) | 0.35 | (0.30, 0.40) | 1.07 | (1.04, 1.10) |
| *<2 years* | 0.24 | (0.03, 1.72) | | 0.31 | (0.08, 1.25) | 1.20 | (0.84, 1.71) | 0.17 | (0.11, 0.27) | 0.96 | (0.89, 1.04) |
| *2-5 years* | 0.81 | (0.38, 1.70) | | 0.44 | (0.20, 0.97) | 1.61 | (1.31, 1.99) | 0.20 | (0.15, 0.27) | 0.99 | (0.94, 1.05) |
| *6-10 years* | 1.59 | (0.88, 2.86) | | 0.36 | (0.14, 0.95) | 1.28 | (0.99, 1.66) | 0.33 | (0.25, 0.45) | 1.07 | (1.01, 1.14) |
| *>10 years* | 1.03 | (0.57, 1.86) | | 0.79 | (0.47, 1.34) | 1.44 | (1.18, 1.75) | 0.60 | (0.50, 0.72) | 1.19 | (1.14, 1.24) |
|  |  |  | |  |  |  |  |  |  |  |  |
| **South Asia** | 1.74 | (1.54, 1.96) | | 0.68 | (0.59, 0.79) | 2.85 | (2.74, 2.97) | 0.16 | (0.15, 0.18) | 0.97 | (0.95, 0.98) |
| *<2 years* | 0.82 | (0.50, 1.34) | | 0.29 | (0.15, 0.55) | 2.56 | (2.30, 2.85) | 0.12 | (0.10, 0.16) | 0.80 | (0.77, 0.84) |
| *2-5 years* | 1.62 | (1.34, 1.97) | | 0.49 | (0.38, 0.64) | 3.05 | (2.88, 3.22) | 0.11 | (0.09, 0.13) | 0.91 | (0.89, 0.93) |
| *6-10 years* | 1.96 | (1.63, 2.35) | | 0.70 | (0.55, 0.88) | 3.00 | (2.83, 3.17) | 0.20 | (0.17, 0.23) | 1.07 | (1.04, 1.09) |
| *>10 years* | 2.08 | (1.64, 2.63) | | 1.23 | (0.97, 1.56) | 2.37 | (2.18, 2.58) | 0.29 | (0.25, 0.34) | 1.04 | (1.01, 1.07) |
|  |  |  | |  |  |  |  |  |  |  |  |
| **South-East/East Asia** | 0.98 | (0.84, 1.14) | | 0.49 | (0.41, 0.57) | 0.99 | (0.93, 1.05 ) | 0.22 | (0.21, 0.24) | 0.46 | (0.45, 0.47) |
| *<2 years* | 0.79 | (0.43, 1.48) | | 0.45 | (0.23, 0.86) | 0.78 | (0.61, 1.00) | 0.15 | (0.11, 0.20) | 0.40 | (0.36, 0.44) |
| *2-5 years* | 0.66 | (0.46, 0.95) | | 0.48 | (0.34, 0.67) | 1.00 | (0.89, 1.13) | 0.18 | (0.15, 0.21) | 0.42 | (0.40, 0.44) |
| *6-10 years* | 1.06 | (0.81, 1.38) | | 0.37 | (0.26, 0.52) | 1.04 | (0.94, 1.16) | 0.19 | (0.16, 0.22) | 0.43 | (0.41, 0.45) |
| *>10 years* | 1.17 | (0.95, 1.44) | | 0.59 | (0.47, 0.74) | 0.97 | (0.89, 1.06) | 0.31 | (0.28, 0.35) | 0.53 | (0.51, 0.55) |
|  |  |  | |  |  |  |  |  |  |  |  |
|  |  |  |  |  |  |  |  |  |  |  |  |
| **Europe** | 0.54 | (0.40, 0.74) | | 0.46 | (0.35, 0.60) | 1.35 | (1.25, 1.4) | 0.58 | (0.53, 0.62) | 0.75 | (0.73, 0.77) |
| *<2 years* | 0.65 | (0.16, 2.59) | | 0.39 | (0.10, 1.56) | 1.19 | (0.80, 1.77) | 0.41 | (0.28, 0.62) | 0.60 | (0.52, 0.70) |
| *2-5 years* | 0.60 | (0.31, 1.15) | | 0.41 | (0.22, 0.76) | 1.39 | (1.18, 1.64) | 0.48 | (0.41, 0.57) | 0.59 | (0.55, 0.63) |
| *6-10 years* | 0.29 | (0.13, 0.64) | | 0.43 | (0.26, 0.72) | 1.46 | (1.27, 1.67) | 0.49 | (0.42, 0.57) | 0.75 | (0.71, 0.79) |
| *>10 years* | 0.73 | (0.48, 1.09) | | 0.53 | (0.37, 0.77) | 1.26 | (1.12, 1.42) | 0.72 | (0.65, 0.79) | 0.86 | (0.83, 0.89) |
|  |  |  | |  |  |  |  |  |  |  |  |
| **Other** | 0.52 | (0.32, 0.85) | | 0.62 | (0.43, 0.88) | 1.45 | (1.28, 1.63) | 0.46 | (0.41, 0.53) | 0.82 | (0.79, 0.85) |
| *<2 years* | Non-estimable^a^ | | | 0.68 | (0.17, 2.69) | 1.51 | (0.96, 2.39) | 0.31 | (0.17, 0.57) | 0.71 | (0.60, 0.84) |
| *2-5 years* | 0.13^a^ | (0.02, 0.94) | | 0.32 | (0.12, 0.86) | 1.48 | (1.18, 1.86) | 0.37 | (0.28, 0.49) | 0.71 | (0.65, 0.77) |
| *6-10 years* | 0.85^a^ | (0.40, 1.78) | | 0.73 | (0.39, 1.36) | 1.75 | (1.44, 2.13) | 0.31 | (0.22, 0.42) | 0.79 | (0.73, 0.85) |
| *>10 years* | 0.69^a^ | (0.34, 1.38) | | 0.77 | (0.46, 1.28) | 1.16 | (0.94, 1.43) | 0.69 | (0.58, 0.82) | 0.96 | (0.91, 1.02) |

^a^ Pre-existing diabetes (for women born in Oceania and Other regions) and pre-existing hypertension (for women born in Oceania) were extremely rare in one or more groups, leading to non-estimable RRs in Poisson/GEE models. We estimated the RR for categories of duration where cell counts were > 0.

^b^ Having ever smoked (smoker or quit <20 weeks gestation) - non-smoker as the reference

**Table S2.** The association between duration of residence and gestational week of first antenatal care attendance, overall and stratified by maternal region of birth, compared with women born in Australia and first attending antenatal care <12 weeks’ gestation. Adjusted relative risks (aRR) and 95% confidence intervals (CI) from robust Poisson^a^ regression models (adjusted for maternal age and year of birth of the child) (*N* = 199,935).

|  | **Gestational week of first antenatal care attendance**  **(ref= <12 weeks)** | | | | | |
| --- | --- | --- | --- | --- | --- | --- |
|  | **12-17 weeks** | | **≥18 weeks** | | **None/Inadequately defined** | |
|  | **aRR** | **95% CI** | **aRR** | **95% CI** | **aRR** | **95% CI** |
| ***Duration of residence*** |  |  |  |  |  |  |
| Australia born | 1.00 (reference) |  | 1.00 (reference) |  | 1.00 (reference) |  |
| < 2 years | 1.29 | (1.24, 1.34) | 2.51 | (2.41, 2.61) | 1.54 | (1.08, 2.19) |
| 2-5 years | 1.38 | (1.35, 1.41) | 1.68 | (1.63, 1.74) | 0.88 | (0.69, 1.13) |
| 6-10 years | 1.32 | (1.29, 1.35) | 1.49 | (1.44, 1.54) | 0.58 | (0.43, 0.77) |
| > 10 years | 1.23 | (1.20, 1.26) | 1.32 | (1.27, 1.37) | 0.95 | (0.76, 1.18) |
|  |  |  |  |  |  |  |
| ***Duration of residence stratified by region of birth*** | | |  |  |  |  |
| **Australia born** | 1.00 (reference) |  | 1.00 (reference) |  | 1.00 (reference) |  |
|  |  |  |  |  |  |  |
| **New Zealand** | 1.62 | (1.57, 1.68) | 2.71 | (2.60, 2.83) | 1.97 | (1.40, 2.78) |
| *<2 years* | 1.62 | (1.31, 1.99) | 3.07 | (2.59, 3.65) | 2.11 | (0.30, 14.88) |
| *2-5 years* | 1.45 | (1.31, 1.61) | 2.57 | (2.31, 2.86) | 2.12 | (0.89, 5.06) |
| *6-10 years* | 1.34 | (1.23, 1.46) | 1.83 | (1.63, 2.05) | 0.92 | (0.35, 2.46) |
| *>10 years* | 1.26 | (1.17, 1.35) | 1.89 | (1.74, 2.06) | 0.68 | (0.28, 1.64) |
|  |  |  |  |  |  |  |
| **Oceania** | 1.20 | (1.16, 1.25) | 1.29 | (1.22, 1.37) | 0.85 | (0.58, 1.13) |
| *<2 years* | 1.35 | (0.92, 1.98) | 4.19 | (3.59, 4.89) | 8.91 | (2.37, 33.41) |
| *2-5 years* | 1.58 | (1.36, 1.85) | 2.76 | (2.37, 3.20) | 3.32 | (1.09, 10.16) |
| *6-10 years* | 1.35 | (1.14, 1.61) | 2.75 | (2.35, 3.21) | 1.99 | (0.50, 7.88) |
| *>10 years* | 1.27 | (1.10,1.45) | 2.15 | (1.85, 2.50) | 3.72 | (1.78, 7.77) |
|  |  |  |  |  |  |  |
| **Africa** | 1.33 | (1.27, 1.40) | 2.07 | (1.95, 2.19) | 1.04 | (0.62, 1.73) |
| *<2 years* | 1.76 | (1.55, 2.00) | 3.55 | (3.19, 3.94) | 2.05 | (0.52, 8.14) |
| *2-5 years* | 1.61 | (1.49, 1.74) | 2.74 | (2.52, 2.98) | 2.77 | (1.50, 5.14) |
| *6-10 years* | 1.69 | (1.58, 1.80) | 2.86 | (2.66, 3.09) | 1.43 | (0.64, 3.17) |
| *>10 years* | 1.57 | (1.49, 1.65) | 2.46 | (2.30, 2.62) | 1.90 | (1.16, 3.12) |
|  |  |  |  |  |  |  |
| **West Asia** | 1.37 | (1.26, 1.50) | 2.67 | (2.46, 2.89) | 3.50 | (2.07, 5.90) |
| *<2 years* | 1.13 | (0.98, 1.31) | 2.27 | (2.01, 2.57) | 2.32 | (0.97, 5.57) |
| *2-5 years* | 1.21 | (1.10, 1.33) | 1.48 | (1.31, 1.68) | 0.21 | (0.03, 1.49) |
| *6-10 years* | 1.16 | (1.04, 1.29) | 1.42 | (1.21, 1.66) | 0.28 | (0.04, 2.01) |
| *>10 years* | 1.19 | (1.09, 1.30) | 1.43 | (1.26, 1.63) | 1.54 | (0.77, 3.08) |
|  |  |  |  |  |  |  |
| **South Asia** | 1.23 | (1.17, 1.29) | 1.76 | (1.64, 1.88) | 1.56 | (1.05, 2.33) |
| *<2 years* | 1.29 | (1.22, 1.37) | 2.20 | (2.07, 2.34) | 0.60 | (0.27, 1.34) |
| *2-5 years* | 1.39 | (1.35, 1.43) | 1.44 | (1.37, 1.51) | 0.57 | (0.37, 0.87) |
| *6-10 years* | 1.38 | (1.33, 1.42) | 1.23 | (1.16, 1.31) | 0.26 | (0.13, 0.52) |
| *>10 years* | 1.25 | (1.20, 1.32) | 1.04 | (0.94, 1.14) | 0.54 | (0.28, 1.05) |
|  |  |  |  |  |  |  |
| **South-East/ East Asia** | 1.23 | (1.16, 1.29) | 1.68 | (1.56, 1.81) | 1.20 | (0.73, 1.97) |
| *<2 years* | 1.24 | (1.14, 1.35) | 2.70 | (2.52, 2.90) | 2.60 | (1.54, 4.40) |
| *2-5 years* | 1.34 | (1.29, 1.39) | 1.66 | (1.57, 1.76) | 0.92 | (0.58, 1.44) |
| *6-10 years* | 1.24 | (1.19, 1.28) | 1.39 | (1.31, 1.48) | 0.53 | (0.31, 0.90) |
| *>10 years* | 1.16 | (1.13, 1.21) | 1.02 | (0.96, 1.09) | 0.80 | (0.55, 1.14) |
|  |  |  |  |  |  |  |
| **Europe** | 1.36 | (1.33, 1.39) | 1.42 | (1.37, 1.47) | 0.47 | (0.35, 0.64) |
| *<2 years* | 1.23 | (1.04, 1.45) | 2.44 | (2.08, 2.87) | 2.24 | (0.73, 6.88) |
| *2-5 years* | 1.31 | (1.22, 1.40) | 1.60 | (1.44, 1.77) | 0.71 | (0.29, 1.70) |
| *6-10 years* | 1.25 | (1.18, 1.33) | 1.32 | (1.18, 1.46) | 1.02 | (0.54, 1.90) |
| *>10 years* | 1.12 | (1.07, 1.19) | 0.97 | (0.87, 1.07) | 0.68 | (0.37, 1.23) |
|  |  |  |  |  |  |  |
| **Other** | 1.18 | (1.12, 1.25) | 1.59 | (1.48, 1.70) | 0.96 | (0.58, 1.60) |
| *<2 years* | 1.17 | (0.92, 1.47) | 2.85 | (2.39, 3.40) | Non-estimable^a^ | |
| *2-5 years* | 1.31 | (1.18, 1.44) | 2.16 | (1.93, 2.43) | 1.82^a^ | (0.82, 4.04) |
| *6-10 years* | 1.11 | (1.00, 1.24) | 1.40 | (1.20, 1.63) | 0.74^a^ | (0.24, 2.30) |
| *>10 years* | 1.26 | (1.16, 1.36) | 1.23 | (1.06, 1.43) | 1.30^a^ | (0.62, 2.73) |

^a^ Among women born in ‘Other’ regions, having ‘None/inadequately defined’ antenatal care was extremely rare in one or more groups, leading to non-estimable RRs in Poisson/GEE models. We estimated the RR for categories of duration where cell counts were > 0.

**Table S3**. The association between duration of residence and pregnancy and birth outcomes (ref = no) stratified by maternal region of birth, compared with women born in Australia. Adjusted relative risks (aRR) and 95% confidence intervals (CI) from robust Poisson^a^ regression models (adjusted for maternal age and year of birth of the child) (*N* =199,935).

|  | **Stillbirth** | | **Preeclampsia** | | **Preterm birth** | | **Gestational diabetes mellitus** | |
| --- | --- | --- | --- | --- | --- | --- | --- | --- |
|  | **aRR** | **95% CI** | **aRR** | **95% CI** | **aRR** | **95% CI** | **aRR** | **95% CI** |
| ***Duration of residence stratified by region of birth*** | |  |  |  |  |  |  |  |
| **Australia born** | 1.00 (reference) |  | 1.00 (reference) |  | 1.00 (reference) |  | 1.00 (reference) |  |
|  |  |  |  |  |  |  |  |  |
| **New Zealand** | 1.49 | (0.95, 2.33) | 1.17 | (0.96, 1.41) | 0.87 | (0.76, 0.99) | 1.28 | (1.18, 1.40) |
| <2 years | 1.63 | (0.23, 11.65) | 1.13 | (0.48, 2.68) | 0.80 | (0.42, 1.51) | 1.44 | (0.98, 2.12) |
| 2-5 years | 0.80 | (0.20, 3.23) | 1.15 | (0.75, 1.78) | 1.14 | (0.88, 1.48) | 1.21 | (0.98, 1.48) |
| 6-10 years | 1.57 | (0.70, 3.51) | 0.84 | (0.56, 1.28) | 0.75 | (0.58, 0.97) | 1.09 | (0.92, 1.29) |
| >10 years | 1.67 | (0.92, 3.04) | 1.36 | (1.06, 1.76) | 0.84 | (0.69, 1.01) | 1.40 | (1.25, 1.57) |
|  |  |  |  |  |  |  |  |  |
| **Oceania** | 1.68 | (0.80, 3.54) | 1.51 | (1.12, 2.03) | 0.86 | (0.68, 1.08) | 2.41 | (2.18, 2.67) |
| <2 years | Non-estimable^a^ | | 1.60 | (0.61, 4.21) | 0.77 | (0.33, 1.80) | 2.28 | (1.56, 3.34) |
| 2-5 years | Non-estimable^a^ | | 0.81 | (0.36, 1.79) | 0.71 | (0.43, 1.19) | 2.28 | (1.84, 2.82) |
| 6-10 years | 1.85^a^ | (0.46, 7.40) | 1.64 | (0.94, 2.86) | 1.00 | (0.65, 1.53) | 2.56 | (2.11, 3.10) |
| >10 years | 2.89^a^ | (1.20, 6.95) | 1.85 | (1.21, 2.81) | 0.87 | (0.61, 1.25) | 2.40 | (2.05, 2.80) |
|  |  |  |  |  |  |  |  |  |
| **Africa** | 2.29 | (1.69, 3.11) | 0.88 | (0.74, 1.06) | 0.90 | (0.81, 1.01) | 1.44 | (1.35, 1.54) |
| <2 years | 2.93 | (1.10, 7.79) | 1.21 | (0.69, 2.13) | 0.81 | (0.53, 1.23) | 1.17 | (0.88, 1.55) |
| 2-5 years | 1.73 | (0.82, 3.65) | 0.85 | (0.57, 1.26) | 0.62 | (0.47, 0.83) | 1.33 | (1.15, 1.54) |
| 6-10 years | 1.79 | (0.92, 3.45) | 0.63 | (0.41, 0.96) | 0.84 | (0.68, 1.05) | 1.33 | (1.16, 1.52) |
| >10 years | 2.71 | (1.82, 4.05) | 0.99 | (0.77, 1.27) | 1.08 | (0.94, 1.24) | 1.58 | (1.45, 1.72) |
|  |  |  |  |  |  |  |  |  |
| **West Asia** | 0.94 | (0.53, 1.67) | 0.52 | (0.39, 0.70) | 0.75 | (0.64, 0.86) | 1.88 | (1.76, 2.02) |
| <2 years | Non-estimable^a^ | | 0.21 | (0.07, 0.66) | 0.91 | (0.65, 1.27) | 1.57 | (1.28, 1.93) |
| 2-5 years | 1.27^a^ | (0.53, 3.05) | 0.51 | (0.30, 0.85) | 0.77 | (0.59, 0.99) | 1.91 | (1.68, 2.16) |
| 6-10 years | Non-estimable^a^ | | 0.73 | (0.43, 1.23) | 0.53 | (0.37, 0.76) | 1.85 | (1.60, 2.14) |
| >10 years | 1.67^a^ | (0.79, 3.52) | 0.54 | (0.33, 0.89) | 0.78 | (0.61, 1.00) | 1.98 | (1.77, 2.21) |
|  |  |  |  |  |  |  |  |  |
| **South Asia** | 1.30 | (1.05, 1.61) | 0.78 | (0.70, 0.86) | 1.07 | (1.02, 1.13) | 2.81 | (2.74, 2.89) |
| <2 years | 1.77 | (1.07, 2.93) | 0.89 | (0.69, 1.15) | 1.06 | (0.92, 1.22) | 2.33 | (2.16, 2.51) |
| 2-5 years | 0.98 | (0.67, 1.42) | 0.77 | (0.66, 0.90) | 0.96 | (0.89, 1.05) | 2.82 | (2.72, 2.93) |
| 6-10 years | 1.58 | (1.15, 2.19) | 0.71 | (0.59, 0.86) | 1.16 | (1.07, 1.26) | 2.96 | (2.85, 3.08) |
| >10 years | 1.27 | (0.78, 2.07) | 0.84 | (0.66, 1.06) | 1.16 | (1.03, 1.30) | 2.79 | (2.65, 2.94) |
|  |  |  |  |  |  |  |  |  |
| **South-East/East Asia** | 0.84 | (0.66, 1.09) | 0.58 | (0.52, 0.66) | 0.80 | (0.75, 0.85) | 2.01 | (1.95, 2.07) |
| <2 years | 0.75 | (0.28, 2.00) | 0.48 | (0.31, 0.77) | 0.65 | (0.52, 0.83) | 1.80 | (1.61, 2.00) |
| 2-5 years | 0.94 | (0.58, 1.53) | 0.49 | (0.38, 0.63) | 0.85 | (0.76, 0.96) | 2.02 | (1.91, 2.14) |
| 6-10 years | 0.99 | (0.64, 1.53) | 0.55 | (0.44, 0.68) | 0.80 | (0.72, 0.90) | 2.05 | (1.95, 2.15) |
| >10 years | 0.71 | (0.46, 1.10) | 0.70 | (0.59, 0.84) | 0.80 | (0.73, 0.88) | 2.02 | (1.94, 2.11) |
|  |  |  |  |  |  |  |  |  |
| **Europe** | 0.92 | (0.62, 1.37) | 0.58 | (0.47, 0.70) | 0.71 | (0.64, 0.79) | 0.98 | (0.92, 1.05) |
| <2 years | 1.56 | (0.39, 6.24) | 0.37 | (0.12, 1.13) | 0.30 | (0.14, 0.62) | 1.04 | (0.77, 1.41) |
| 2-5 years | 1.01 | (0.45, 2.26) | 0.51 | (0.33, 0.78) | 0.73 | (0.59, 0.90) | 0.83 | (0.71, 0.97) |
| 6-10 years | 0.76 | (0.34, 1.70) | 0.53 | (0.36, 0.77) | 0.71 | (0.59, 0.86) | 0.94 | (0.83, 1.06) |
| >10 years | 0.96 | (0.54, 1.71) | 0.70 | (0.53, 0.91) | 0.77 | (0.66, 0.89) | 1.10 | (1.01, 1.21) |
|  |  |  |  |  |  |  |  |  |
| **Other** | 0.61 | (0.29, 1.28) | 0.78 | (0.60, 1.01) | 0.81 | (0.70, 0.94) | 1.02 | (0.92, 1.12) |
| <2 years | 1.35 | (0.19, 9.58) | 1.00 | (0.42, 2.39) | 0.29 | (0.11, 0.76) | 0.74 | (0.46, 1.18) |
| 2-5 years | 0.33 | (0.05, 2.36) | 0.79 | (0.49, 1.29) | 0.87 | (0.67, 1.14) | 0.97 | (0.79, 1.18) |
| 6-10 years | 0.63 | (0.16, 2.54) | 0.66 | (0.38, 1.13) | 0.87 | (0.66, 1.13) | 0.97 | (0.80, 1.17) |
| >10 years | 0.68 | (0.22, 2.10) | 0.85 | (0.56, 1.27) | 0.84 | (0.66, 1.05) | 1.16 | (1.00, 1.34) |

^a^ For women born in Oceania and West Asia, stillbirths were extremely rare in one or more groups, leading to non-estimable RRs in Poisson/GEE models. We estimated the RR for categories of duration where cell counts were > 0.

**Table S4. Sensitivity analysis 1:** The association between duration of residence and preconception health factors (ref = no), stratified by maternal region of birth, compared with women born in Australia. Adjusted relative risks (aRR) and 95% confidence intervals (CI) from robust Poisson^a^ regression models (adjusted for maternal age and year of birth of the child). Results from models in a **sensitivity analysis including women with missing duration as a category,** but excluding observations missing information on smoking and BMI (N=212,214).

|  | **Stillbirth** | | **Preeclampsia** | | **Preterm birth** | | **Gestational diabetes mellitus** | |
| --- | --- | --- | --- | --- | --- | --- | --- | --- |
|  | **aRR** | **95% CI** | **aRR** | **95% CI** | **aRR** | **95% CI** | **aRR** | **95% CI** |
| ***Duration of residence stratified*** |  |  |  |  |  |  |  |  |
| Australia born | 1.00 (reference) |  | 1.00 (reference) |  | 1.00 (reference) |  | 1.00 (reference) |  |
| < 2 years | 1.35 | (0.92, 1.98) | 0.75 | (0.62, 0.91) | 0.85 | (0.76, 0.94) | 1.89 | (1.78, 2.00) |
| 2-5 years | 0.97 | (0.75, 1.25) | 0.69 | (0.61, 0.77) | 0.89 | (0.84, 0.94) | 2.16 | (2.09, 2.22) |
| >5-10 years | 1.21 | (0.96, 1.53) | 0.65 | (0.58, 0.73) | 0.91 | (0.86, 0.97) | 2.09 | (2.03, 2.16) |
| > 10 years | 1.23 | (0.99, 1.52) | 0.82 | (0.74, 0.91) | 0.88 | (0.83, 0.93) | 1.87 | (1.82, 1.93) |
| Missing | 1.83 | (1.44, 2.34) | 0.87 | (0.77, 1.00) | 1.24 | (1.17, 1.33) | 1.76 | (1.68, 1.83) |
|  |  |  |  |  |  |  |  |  |
| ***Duration of residence stratified by region of birth*** | |  |  |  |  |  |  |  |
| **Australia born** | 1.00 (reference) |  | 1.00 (reference) |  | 1.00 (reference) |  | 1.00 (reference) |  |
|  |  |  |  |  |  |  |  |  |
| **New Zealand** | 1.42 | (0.94, 2.17) | 1.13 | (0.95, 1.35) | 0.90 | (0.80, 1.01) | 1.21 | (1.12, 1.31) |
| <2 years | 1.63 | (0.23, 11.64) | 1.13 | (0.48, 2.68) | 0.80 | (0.42, 1.51) | 1.44 | (0.98, 2.12) |
| 2-5 years | 0.80 | (0.20, 3.23) | 1.15 | (0.75, 1.78) | 1.14 | (0.88, 1.48) | 1.21 | (0.98, 1.48) |
| >5-10 years | 1.57 | (0.70, 3.51) | 0.84 | (0.56, 1.28) | 0.75 | (0.58, 0.97) | 1.09 | (0.92, 1.29) |
| >10 years | 1.67 | (0.92, 3.03) | 1.37 | (1.06, 1.76) | 0.83 | (0.69, 1.01) | 1.40 | (1.25, 1.57) |
| Missing | 1.08 | (0.35, 3.34) | 0.95 | (0.59, 1.52) | 1.04 | (0.80, 1.35) | 0.85 | (0.67, 1.07) |
|  |  |  |  |  |  |  |  |  |
| **Oceania** | 1.87 | (0.97, 3.60) | 1.58 | (1.21, 2.07) | 0.95 | (0.77, 1.17) | 2.42 | (2.20, 2.66) |
| <2 years | Non-estimable^a^ | | 1.60 | (0.61, 4.21) | 0.77 | (0.33, 1.80) | 2.28 | (1.56, 3.34) |
| 2-5 years | Non-estimable^a^ | | 0.81 | (0.36, 1.79) | 0.71 | (0.43, 1.19) | 2.28 | (1.84, 2.82) |
| >5-10 years | 1.85 | (0.46, 7.40) | 1.64 | (0.94, 2.86) | 1.00 | (0.65, 1.53) | 2.56 | (2.11, 3.10) |
| >10 years | 2.89 | (1.20, 6.95) | 1.85 | (1.21, 2.81) | 0.87 | (0.61, 1.25) | 2.40 | (2.05, 2.80) |
| Missing | 2.90 | (0.72, 11.57) | 2.08 | (1.10, 3.94) | 1.55 | (1.01, 2.37) | 2.50 | (1.96, 3.19) |
|  |  |  |  |  |  |  |  |  |
| **Africa** | 2.44 | (1.85, 3.21) | 0.88 | (0.74, 1.04) | 0.97 | (0.89, 1.07) | 1.43 | (1.35, 1.52) |
| <2 years | 2.93 | (1.10, 7.81) | 1.22 | (0.69, 2.13) | 0.81 | (0.53, 1.23) | 1.17 | (0.88, 1.55) |
| 2-5 years | 1.73 | (0.82, 3.65) | 0.85 | (0.57, 1.26) | 0.62 | (0.47, 0.83) | 1.33 | (1.15, 1.54) |
| >5-10 years | 1.79 | (0.92, 3.45) | 0.63 | (0.41, 0.96) | 0.84 | (0.68, 1.05) | 1.33 | (1.16, 1.52) |
| >10 years | 2.71 | (1.82, 4.05) | 0.99 | (0.77, 1.27) | 1.08 | (0.94, 1.24) | 1.58 | (1.45, 1.72) |
| Missing | 3.22 | (1.82, 5.70) | 0.84 | (0.55, 1.28) | 1.34 | (1.10, 1.63) | 1.37 | (1.18, 1.59) |
|  |  |  |  |  |  |  |  |  |
| **West Asia** | 1.15 | (0.72, 1.84) | 0.55 | (0.43, 0.71) | 0.85 | (0.76, 0.96) | 1.89 | (1.78, 2.02) |
| <2 years | Non-estimable^a^ | | 0.21 | (0.07, 0.66) | 0.91 | (0.65, 1.27) | 1.57 | (1.28, 1.93) |
| 2-5 years | 1.27^a^ | (0.53, 3.06) | 0.51 | (0.30, 0.85) | 0.77 | (0.59, 0.99) | 1.91 | (1.68, 2.16) |
| >5-10 years | Non-estimable^a^ | | 0.73 | (0.43, 1.23) | 0.53 | (0.37, 0.76) | 1.85 | (1.60, 2.14) |
| >10 years | 1.67^a^ | (0.79, 3.52) | 0.54 | (0.33, 0.89) | 0.78 | (0.61, 1.00) | 1.98 | (1.77, 2.21) |
| Missing | 2.00^a^ | (0.89, 4.47) | 0.68 | (0.40, 1.15) | 1.31 | (1.05, 1.64) | 1.93 | (1.67, 2.22) |
|  |  |  |  |  |  |  |  |  |
| **South Asia** | 1.43 | (1.18, 1.74) | 0.79 | (0.72, 0.87) | 1.11 | (1.05, 1.16) | 2.81 | (2.74, 2.88) |
| <2 years | 1.77 | (1.07, 2.93) | 0.89 | (0.69, 1.15) | 1.06 | (0.92, 1.22) | 2.33 | (2.17, 2.51) |
| 2-5 years | 0.97 | (0.67, 1.41) | 0.77 | (0.66, 0.90) | 0.96 | (0.89, 1.05) | 2.82 | (2.72, 2.93) |
| >5-10 years | 1.58 | (1.15, 2.18) | 0.71 | (0.59, 0.86) | 1.16 | (1.07, 1.26) | 2.96 | (2.85, 3.08) |
| >10 years | 1.27 | (0.78, 2.06) | 0.83 | (0.66, 1.06) | 1.15 | (1.03, 1.29) | 2.79 | (2.65, 2.94) |
| Missing | 2.26 | (1.55, 3.28) | 0.84 | (0.67, 1.06) | 1.31 | (1.18, 1.46) | 2.74 | (2.60, 2.90) |
|  |  |  |  |  |  |  |  |  |
| **South-East/East Asia** | 0.84 | (0.66, 1.08) | 0.61 | (0.55, 0.68) | 0.85 | (0.80, 0.90) | 1.96 | (1.90, 2.02) |
| <2 years | 0.75 | (0.28, 2.00) | 0.48 | (0.31, 0.77) | 0.65 | (0.52, 0.83) | 1.80 | (1.61, 2.00) |
| 2-5 years | 0.94 | (0.58, 1.52) | 0.49 | (0.38, 0.63) | 0.85 | (0.76, 0.96) | 2.02 | (1.91, 2.14) |
| >5-10 years | 0.99 | (0.64, 1.53) | 0.55 | (0.44, 0.68) | 0.80 | (0.72, 0.90) | 2.05 | (1.95, 2.15) |
| >10 years | 0.71 | (0.46, 1.10) | 0.70 | (0.59, 0.83) | 0.80 | (0.73, 0.88) | 2.02 | (1.94, 2.11) |
| Missing | 0.94 | (0.50, 1.75) | 0.83 | (0.54, 1.08) | 1.20 | (1.06, 1.36) | 1.62 | (1.50, 1.76) |
|  |  |  |  |  |  |  |  |  |
| **Europe** | 1.04 | (0.74, 1.47) | 0.65 | (0.55, 0.78) | 0.75 | (0.68, 0.82) | 0.93 | (0.87, 0.99) |
| <2 years | 1.56 | (0.39, 6.24) | 0.37 | (0.12, 1.13) | 0.30 | (0.14, 0.62) | 1.04 | (0.77, 1.41) |
| 2-5 years | 1.01 | (0.45, 2.26) | 0.51 | (0.33, 0.78) | 0.73 | (0.59, 0.90) | 0.83 | (0.71, 0.97) |
| >5-10 years | 0.76 | (0.34, 1.70) | 0.53 | (0.36, 0.77) | 0.71 | (0.59, 0.86) | 0.94 | (0.83, 1.06) |
| >10 years | 0.96 | (0.54, 1.70) | 0.70 | (0.53, 0.91) | 0.77 | (0.66, 0.89) | 1.10 | (1.01, 1.21) |
| Missing | 1.72 | (0.89, 3.32) | 1.12 | (0.81, 1.55) | 0.99 | (0.81, 1.20) | 0.66 | (0.55, 0.79) |
|  |  |  |  |  |  |  |  |  |
| **Other** | 0.86 | (0.48, 1.52) | 0.76 | (0.59, 0.96) | 0.93 | (0.82, 1.06) | 0.98 | (0.89, 1.07) |
| <2 years | 1.35 | (0.19, 9.58) | 1.00 | (0.42, 2.39) | 0.29 | (0.11, 0.76) | 0.74 | (0.46, 1.18) |
| 2-5 years | 0.33 | (0.05, 2.36) | 0.79 | (0.49, 1.29) | 0.87 | (0.67, 1.14) | 0.97 | (0.79, 1.18) |
| >5-10 years | 0.63 | (0.16, 2.53) | 0.66 | (0.38, 1.13) | 0.87 | (0.66, 1.13) | 0.97 | (0.80, 1.17) |
| >10 years | 0.67 | (0.22, 2.09) | 0.85 | (0.56, 1.27) | 0.84 | (0.66, 1.05) | 1.16 | (1.00, 1.34) |
| Missing | 2.11 | (0.88, 5.08) | 0.68 | (0.37, 1.26) | 1.57 | (1.25, 1.97) | 0.80 | (0.62, 1.03) |

^a^ For women born in Oceania and West Asia, stillbirths were extremely rare in one or more groups, leading to non-estimable RRs in Poisson/GEE models. We estimated the RR for categories of duration where cell counts were > 0.

**Table S5. Sensitivity analysis 1:** The association between duration of residence and gestational week of first antenatal care attendance, overall and stratified by maternal region of birth, compared with women born in Australia and first attending antenatal care <12 weeks’ gestation. Adjusted relative risks (aRR) and 95% confidence intervals (CI) from robust Poisson^a^ regression models (adjusted for maternal age and year of birth of the child). Results from models in a **sensitivity analysis including women with missing duration as a category,** excluding observations missing information on smoking and BMI (N=212,214).

|  | **Pre-existing diabetes** | | | **Pre-existing hypertension** | | **Pre-existing thyroid condition** | | **Smoking^b^** | | **Early-pregnancy weight status BMI ≥25 kg/m^2^** | |
| --- | --- | --- | --- | --- | --- | --- | --- | --- | --- | --- | --- |
|  | **aRR** | **95% CI** | | **aRR** | **95% CI** | **aRR** | **95% CI** | **aRR** | **95% CI** | **aRR** | **95% CI** |
| ***Duration of residence*** | |  | |  |  |  |  |  |  |  |  |
| Australia born | 1.00 (reference) |  | | 1.00 (reference) |  | 1.00 (reference) |  | 1.00 (reference) |  | 1.00 (reference) |  |
| *<2 years* | 0.74 | (0.52, 1.04) | | 0.48 | (0.34, 0.67) | 1.63 | (1.49, 1.78) | 0.20 | (0.17, 0.23) | 0.74 | (0.71, 0.76) |
| *2-5 years* | 1.18 | (1.01, 1.37) | | 0.49 | (0.41, 0.58) | 1.99 | (1.90, 2.09) | 0.22 | (0.20, 0.23) | 0.78 | (0.77,0.79) |
| *>5-10 years* | 1.34 | (1.16, 1.54) | | 0.58 | (0.50, 0.68) | 1.84 | (1.76, 1.93) | 0.29 | (0.27, 0.31) | 0.84 | (0.82, 0.85) |
| *>10 years* | 1.24 | (1.09, 1.42) | | 0.78 | (0.69, 0.88) | 1.27 | (1.21, 1.34) | 0.53 | (0.51, 0.56) | 0.85 | (0.84, 0.87) |
| *Missing* | 1.36 | (1.14, 1.62) | | 0.55 | (0.45, 0.68) | 1.55 | (1.46, 1.66) | 0.22 | (0.20, 0.25) | 0.83 | (0.81, 0.84) |
|  |  |  | |  |  |  |  |  |  |  |  |
| ***Duration of residence stratified by region of birth*** | | | | |  |  |  |  |  |  |  |
| **Australia born** | 1.00 (reference) |  | | 1.00 (reference) |  | 1.00 (reference) |  | 1.00 (reference) |  | 1.00 (reference) |  |
|  |  |  | |  |  |  |  |  |  |  |  |
| **New Zealand** | 1.38 | (1.05, 1.82) | | 1.12 | (0.89, 1.42) | 0.78 | (0.68, 0.90) | 1.15 | (1.08, 1.22) | 1.24 | (1.21, 1.27) |
| *<2 years* | 0.80 | (0.11, 5.69) | | 0.53 | (0.07, 3.76) | 0.57 | (0.22, 1.52) | 1.38 | (1.07, 1.78) | 1.47 | (1.37, 1.58) |
| *2-5 years* | 1.78 | (0.96, 3.30) | | 0.45 | (0.17, 1.20) | 0.86 | (0.60, 1.23) | 1.08 | (0.92, 1.27) | 1.28 | (1.22, 1.35) |
| *>5-10 years* | 0.98 | (0.51, 1.89) | | 1.35 | (0.87, 2.09) | 0.79 | (0.59, 1.05) | 1.00 | (0.87, 1.15) | 1.22 | (1.16, 1.27) |
| *>10 years* | 1.45 | (0.96, 2.19) | | 1.28 | (0.91, 1.80) | 0.88 | (0.71, 1.08) | 1.35 | (1.24, 1.48) | 1.27 | (1.23, 1.31) |
| *Missing* | 1.52 | (0.82, 2.81) | | 1.11 | (0.63, 1.94) | 0.51 | (0.34, 0.78) | 0.85 | (0.71, 1.02) | 1.08 | (1.02, 1.15) |
|  |  |  | |  |  |  |  |  |  |  |  |
| **Oceania** | 2.74^a^ | (1.92, 3.90) | | 0.65 | (0.37, 1.14) | 0.89 | (0.69, 1.14) | 0.80 | (0.69, 0.92) | 1.48 | (1.44,1.52) |
| *<2 years* | Non-estimable^a^ | | | 1.64^a^ | (0.42, 6.51) | 0.45 | (0.11, 1.78) | 0.35 | (0.16, 0.75) | 1.51 | (1.37, 1.66) |
| *2-5 years* | 2.56^a^ | (1.16, 5.67) | | 0.54^a^ | (0.14, 2.13) | 0.73 | (0.40, 1.34) | 0.55 | (0.38, 0.78) | 1.54 | (1.46, 1.62) |
| *>5-10 years* | 3.24^a^ | (1.63, 6.44) | | 1.25^a^ | (0.52, 2.97) | 1.35 | (0.88, 2.06) | 0.71 | (0.52, 0.97) | 1.44 | (1.36, 1.53) |
| *>10 years* | 3.07^a^ | (1.79, 5.27) | | Non-estimable^a^ | | 0.80 | (0.53, 1.22) | 1.21 | (1.00, 1.46) | 1.44 | (1.37, 1.51) |
| *Missing* | 2.58^a^ | (0.98, 6.80) | | 1.16^a^ | (0.39, 3.49) | 0.84 | (0.43, 1.66) | 0.58 | (0.36, 0.91) | 1.50 | (1.39, 1.61) |
|  |  |  | |  |  |  |  |  |  |  |  |
| **Africa** | 1.26 | (0.99, 1.60) | | 0.86 | (0.69, 1.08) | 0.95 | (0.85, 1.06) | 0.26 | (0.23, 0.29) | 1.01 | (0.99, 1.04) |
| *<2 years* | 1.01 | (0.33, 3.11) | | 1.51 | (0.72, 3.15) | 1.01 | (0.64, 1.59) | 0.12 | (0.06, 0.23) | 0.86 | (0.78, 0.96) |
| *2-5 years* | 1.36 | (0.79, 2.34) | | 0.65 | (0.35, 1.21) | 1.21 | (0.96, 1.52) | 0.13 | (0.09, 0.20) | 0.98 | (0.93, 1.03) |
| *>5-10 years* | 1.40 | (0.87, 2.26) | | 0.61 | (0.35, 1.08) | 0.97 | (0.77, 1.21) | 0.21 | (0.16, 0.28) | 1.04 | (1.00, 1.09) |
| *>10 years* | 1.22 | (0.84, 1.77) | | 1.11 | (0.82, 1.50) | 0.87 | (0.73, 1.04) | 0.41 | (0.35, 0.48) | 1.05 | (1.01, 1.08) |
| *Missing* | 1.15 | (0.62, 2.13) | | 0.57 | (0.28, 1.13) | 0.81 | (0.60, 1.08) | 0.19 | (0.13, 0.26) | 0.98 | (0.93, 1.04) |
|  |  |  | |  |  |  |  |  |  |  |  |
| **West Asia** | 1.02 | (0.74, 1.41) | | 0.61 | (0.44, 0.85) | 1.38 | (1.24, 1.54) | 0.33 | (0.29, 0.38) | 1.08 | (1.05, 1.10) |
| *<2 years* | 0.24 | (0.03, 1.72) | | 0.31 | (0.08, 1.25) | 1.20 | (0.84, 1.71) | 0.17 | (0.11, 0.27) | 0.96 | (0.89, 1.05) |
| *2-5 years* | 0.81 | (0.38, 1.70) | | 0.44 | (0.20, 0.97) | 1.61 | (1.31, 1.99) | 0.20 | (0.15, 0.27) | 0.99 | (0.94, 1.05) |
| *>5-10 years* | 1.59 | (0.88, 2.87) | | 0.36 | (0.14, 0.95) | 1.28 | (0.99, 1.66) | 0.33 | (0.25, 0.45) | 1.07 | (1.01, 1.14) |
| *>10 years* | 1.03 | (0.57, 1.86) | | 0.79 | (0.47, 1.34) | 1.44 | (1.18, 1.75) | 0.60 | (0.50, 0.72) | 1.19 | (1.14, 1.24) |
| *Missing* | 1.04 | (0.50, 2.19) | | 0.93 | (0.50, 1.72) | 1.22 | (0.93, 1.60) | 0.28 | (0.20, 0.38) | 1.07 | (1.01, 1.14) |
|  |  |  | |  |  |  |  |  |  |  |  |
| **South Asia** | 1.75 | (1.56, 1.97) | | 0.66 | (0.57, 0.75) | 2.85 | (2.74, 2.96) | 0.15 | (0.13, 0.16) | 0.97 | (0.96, 0.98) |
| *<2 years* | 0.83 | (0.50, 1.36) | | 0.29 | (0.15, 0.56) | 2.56 | (2.30, 2.85) | 0.12 | (0.10, 0.16) | 0.81 | (0.77, 0.84) |
| *2-5 years* | 1.63 | (1.34, 1.98) | | 0.49 | (0.38, 0.64) | 3.05 | (2.89, 3.22) | 0.11 | (0.09, 0.13) | 0.91 | (0.89, 0.93) |
| *>5-10 years* | 1.96 | (1.63, 2.62) | | 0.70 | (0.55, 0.88) | 3.00 | (2.84, 3.18) | 0.20 | (0.17, 0.23) | 1.07 | (1.04, 1.09) |
| *>10 years* | 2.07 | (1.63, 2.62) | | 1.23 | (0.97, 1.55) | 2.38 | (2.18, 2.59) | 0.29 | (0.25, 0.34) | 1.04 | (1.01, 1.07) |
| *Missing* | 1.78 | (1.36, 2.33) | | 0.50 | (0.34, 0.74) | 2.80 | (2.58, 3.03) | 0.04 | (0.02, 0.05) | 0.98 | (0.95, 1.01) |
|  |  |  | |  |  |  |  |  |  |  |  |
| **South-East/East Asia** | 1.00 | (0.86, 1.15) | | 0.47 | (0.40, 0.55) | 0.98 | (0.93, 1.04 ) | 0.22 | (0.20, 0.23) | 0.47 | (0.46, 0.48) |
| *<2 years* | 0.80 | (0.43, 1.48) | | 0.45 | (0.23, 0.86) | 0.78 | (0.61, 1.00) | 0.15 | (0.11, 0.20) | 0.40 | (0.36, 0.44) |
| *2-5 years* | 0.66 | (0.46, 0.95) | | 0.48 | (0.34, 0.67) | 1.00 | (0.89, 1.13) | 0.18 | (0.15, 0.21) | 0.42 | (0.40, 0.44) |
| *>5-10 years* | 1.06 | (0.81, 1.38) | | 0.37 | (0.26, 0.52) | 1.04 | (0.94, 1.16) | 0.19 | (0.16, 0.22) | 0.43 | (0.41, 0.45) |
| *>10 years* | 1.17 | (0.94, 1.44) | | 0.59 | (0.47, 0.74) | 0.97 | (0.89, 1.06) | 0.31 | (0.28, 0.34) | 0.53 | (0.51, 0.55) |
| *Missing* | 1.18 | (0.83, 1.67) | | 0.38 | (0.24, 0.61) | 0.91 | (0.78, 1.06) | 0.15 | (0.12 , 0.20) | 0.49 | (0.47, 0.52) |
|  |  |  |  |  |  |  |  |  |  |  |  |
| **Europe** | 0.65 | (0.50, 0.85) | | 0.45 | (0.35, 0.57) | 1.34 | (1.25, 1.45) | 0.55 | (0.51 , 0.59) | 0.73 | (0.71, 0.75) |
| *<2 years* | 0.65 | (0.16, 2.59) | | 0.39 | (0.10, 1.56) | 1.19 | (0.80, 1.77) | 0.41 | (0.28 , 0.62) | 0.60 | (0.52, 0.70) |
| *2-5 years* | 0.60 | (0.31, 1.15) | | 0.41 | (0.22, 0.76) | 1.39 | (1.18, 1.64) | 0.48 | (0.41, 0.57) | 0.59 | (0.55, 0.63) |
| *>5-10 years* | 0.29 | (0.13, 0.64) | | 0.43 | (0.26, 0.72) | 1.46 | (1.27, 1.67) | 0.49 | (0.42, 0.57) | 0.75 | (0.71, 0.78) |
| *>10 years* | 0.73 | (0.48, 1.09) | | 0.53 | (0.37, 0.77) | 1.26 | (1.12, 1.42) | 0.72 | (0.65, 0.80) | 0.86 | (0.83, 0.89) |
| *Missing* | 1.33 | (0.84, 2.11) | | 0.40 | (0.21, 0.76) | 1.27 | (1.05, 1.52) | 0.42 | (0.34, 0.52) | 0.67 | (0.63, 0.72) |
|  |  |  | |  |  |  |  |  |  |  |  |
| **Other** | 0.51 | (0.33, 0.81) | | 0.64 | (0.47, 0.88) | 1.41 | (1.26, 1.57) | 0.44 | (0.39, 0.50) | 0.81 | (0.78, 0.84) |
| *<2 years* | Non-estimable^a^ | | | 0.68 | (0.17, 2.69) | 1.51 | (0.96, 2.39) | 0.31 | (0.17, 0.57) | 0.71 | (0.60, 0.84) |
| *2-5 years* | 0.13^a^ | (0.02, 0.94) | | 0.32 | (0.12, 0.86) | 1.48 | (1.18, 1.86) | 0.37 | (0.28 , 0.49) | 0.71 | (0.65, 0.77) |
| *>5-10 years* | 0.85^a^ | (0.40, 1.78) | | 0.73 | (0.39, 1.36) | 1.75 | (1.44, 2.13) | 0.31 | (0.22, 0.42) | 0.79 | (0.73, 0.85) |
| *>10 years* | 0.69^a^ | (0.34, 1.38) | | 0.77 | (0.46, 1.28) | 1.16 | (0.94, 1.43) | 0.69 | (0.58, 0.82) | 0.96 | (0.91, 1.02) |
| *Missing* | 0.51^a^ | (0.16, 1.56) | | 0.81 | (0.41, 1.62) | 1.24 | (0.94, 1.63) | 0.33 | (0.24, 0.47) | 0.80 | (0.74, 0.88) |

^a^ Pre-existing diabetes (for women born in Oceania and Other regions) and pre-existing hypertension (for women born in Oceania) were extremely rare in one or more groups, leading to non-estimable RRs in Poisson/GEE models. We estimated the RR for categories of duration of residence where cell counts were >0.

^b^ Having ever smoked (smoker or quit <20 weeks gestation) - non-smoker as the reference.

**Table S6. Sensitivity analysis 1:** The association between duration of residence and pregnancy and birth outcomes (ref = no), overall and stratified by maternal region of birth, compared with women born in Australia. Relative risks (aRR) and 95% confidence intervals (CI) from robust Poisson^a^ regression models (adjusted for maternal age and year of birth of the child). Results from models in a **sensitivity analysis including women with missing duration as a category,** excluding observations missing information on smoking and BMI (N=212,214).

|  | **Gestational week of first antenatal care attendance**  **(ref= <12 weeks)** | | |
| --- | --- | --- | --- |
|  | **12-17 weeks** | **≥18 weeks** | **None/Inadequately defined** |
|  | **aRR (95% CI)** | **aRR (95% CI)** | **aRR (95% CI)** |
| ***Duration of residence*** |  |  |  |
| Australia born | 1.00 (reference) | 1.00 (reference) | 1.00 (reference) |
| < 2 years | 1.29 (1.24,1.34) | 2.51 (2.41,2.61) | 1.53 (1.08,2.17) |
| 2-5 years | 1.38 (1.35,1.40) | 1.68 (1.62,1.73) | 0.88 (0.69,1.13) |
| >5-10 years | 1.32 (1.29,1.35) | 1.48 (1.43,1.54) | 0.58 (0.43,0.77) |
| > 10 years | 1.23 (1.20,1.26) | 1.32 (1.27,1.36) | 0.94 (0.76,1.17) |
| Missing | 1.72 (1.68,1.76) | 2.61 (2.53,2.70) | 3.37 (2.77,4.10) |
|  |  |  |  |
| ***Duration of residence stratified by region of birth*** | |  |  |
| **Australia born** | 1.00 (reference) | 1.00 (reference) | 1.00 (reference) |
|  |  |  |  |
| **New Zealand** | 1.68 (1.63,1.73) | 2.86 (2.76,2.97) | 2.49 (1.86,3.32) |
| *<2 years* | 1.61 (1.31,1.99) | 3.08 (2.59,3.65) | 2.11 (0.30,14.87) |
| *2-5 years* | 1.45 (1.31,1.61) | 2.57 (2.31,2.86) | 2.12 (0.89,5.06) |
| *>5-10 years* | 1.34 (1.23,1.46) | 1.83 (1.63,2.05) | 0.92 (0.35,2.46) |
| *>10 years* | 1.26 (1.17,1.35) | 1.89 (1.74,2.06) | 0.68 (0.28,1.64) |
| *Missing* | 1.72 (1.58,1.88) | 2.89 (2.61,3.21) | 4.79 (2.67,8.60) |
|  |  |  |  |
| **Oceania** | 1.27 (1.23,1.31) | 1.47 (1.40,1.55) | 0.97 (0.70,1.35) |
| *<2 years* | 1.35 (0.92,1.98) | 4.19 (3.59,4.89) | 8.87 (2.36,33.26) |
| *2-5 years* | 1.58 (1.36,1.85) | 2.76 (2.37,3.20) | 3.31 (1.08,10.13) |
| *>5-10 years* | 1.35 (1.14,1.61) | 2.75 (2.35,3.21) | 1.98 (0.50,7.86) |
| *>10 years* | 1.27 (1.10,1.45) | 2.15 (1.85,2.50) | 3.72 (1.78,7.77) |
| *Missing* | 1.66 (1.37,2.00) | 2.81 (2.28,3.45) | 8.72 (3.75,20.27) |
|  |  |  |  |
| **Africa** | 1.40 (1.34,1.46) | 2.19 (2.09,2.31) | 1.54 (1.05,2.28) |
| *<2 years* | 1.76 (1.55,2.00) | 3.55 (3.20,3.95) | 2.05 (0.52,8.14) |
| *2-5 years* | 1.61 (1.49,1.74) | 2.74 (2.52,2.98) | 2.77 (1.50,5.14) |
| *>5-10 years* | 1.69 (1.58,1.80) | 2.87 (2.66,3.09) | 1.43 (0.64,3.17) |
| *>10 years* | 1.57 (1.49,1.65) | 2.45 (2.30,2.62) | 1.90 (1.16,3.12) |
| *Missing* | 2.02 (1.89,2.16) | 3.72 (3.47,3.99) | 6.31 (3.83,10.41) |
|  |  |  |  |
| **West Asia** | 1.41 (1.30,1.53) | 2.68 (2.49,2.89) | 4.12 (2.63,6.45) |
| *<2 years* | 1.13 (0.98,1.31) | 2.27 (2.01,2.58) | 2.32 (0.97,5.57) |
| *2-5 years* | 1.21 (1.10,1.32) | 1.49 (1.31,1.68) | 0.21 (0.03,1.49) |
| *>5-10 years* | 1.16 (1.04,1.29) | 1.42 (1.21,1.66) | 0.28 (0.04,2.00) |
| *>10 years* | 1.19 (1.09,1.30) | 1.43 (1.26,1.63) | 1.54 (0.77,3.08) |
| *Missing* | 1.57 (1.43,1.72) | 2.84 (2.58,3.12) | 5.31 (3.15,8.95) |
|  |  |  |  |
| **South Asia** | 1.23 (1.17,1.29) | 1.76 (1.64,1.88) | 1.56 (1.05,2.33) |
| *<2 years* | 1.29 (1.22,1.37) | 2.20 (2.06,2.34) | 0.60 (0.27,1.33) |
| *2-5 years* | 1.39 (1.35,1.43) | 1.43 (1.37,1.51) | 0.56 (0.36,0.87) |
| *>5-10 years* | 1.38 (1.33,1.42) | 1.23 (1.16,1.31) | 0.26 (0.13,0.52) |
| *>10 years* | 1.25 (1.19,1.31) | 1.04 (0.94,1.14) | 0.54 (0.28,1.04) |
| *Missing* | 1.89 (1.83,1.96) | 2.49 (2.35,2.63) | 2.48 (1.69,3.64) |
|  |  |  |  |
| **South-East/ East Asia** | 1.27 (1.24,1.30) | 1.54 (1.49,1.59) | 1.08 (0.88,1.33) |
| *<2 years* | 1.24 (1.14,1.35) | 2.70 (2.51,2.90) | 2.59 (1.53,4.38) |
| *2-5 years* | 1.34 (1.29,1.39) | 1.66 (1.57,1.76) | 0.92 (0.58,1.44) |
| *>5-10 years* | 1.24 (1.19,1.28) | 1.39 (1.31,1.48) | 0.53 (0.31,0.90) |
| *>10 years* | 1.16 (1.12,1.21) | 1.02 (0.96,1.09) | 0.80 (0.55,1.14) |
| *Missing* | 1.57 (1.49,1.64) | 2.38 (2.24,2.54) | 3.01 (2.10,4.32) |
|  |  |  |  |
| **Europe** | 1.43(1.40,1.45) | 1.55(1.50,1.60) | 0.68(0.53,0.87) |
| *<2 years* | 1.23 (1.04,1.45) | 2.44 (2.08,2.87) | 2.23 (0.73,6.88) |
| *2-5 years* | 1.31 (1.22,1.40) | 1.60 (1.44,1.77) | 0.71 (0.29,1.70) |
| *>5-10 years* | 1.25 (1.18,1.33) | 1.32 (1.18,1.46) | 1.02 (0.54,1.89) |
| *>10 years* | 1.12 (1.07,1.19) | 0.97 (0.87,1.07) | 0.67 (0.37,1.22) |
| *Missing* | 1.69 (1.58,1.80) | 2.53 (2.31,2.76) | 1.93 (1.00,3.71) |
|  |  |  |  |
| **Other** | 1.25(1.19,1.30) | 1.81 (1.71,1.92) | 1.58 (1.09,2.29) |
| *<2 years* | 1.17 (0.92,1.47) | 2.85 (2.39,3.40) | Non-estimable^a^ |
| *2-5 years* | 1.31 (1.18,1.44) | 2.16 (1.93,2.43) | 1.81 (0.82,4.04) |
| *>5-10 years* | 1.11 (1.00,1.24) | 1.40 (1.19,1.63) | 0.74 (0.24,2.30) |
| *>10 years* | 1.26 (1.16,1.36) | 1.23 (1.06,1.43) | 1.30 (0.62,2.72) |
| *Missing* | 1.26 (1.12,1.42) | 2.14 (1.87,2.45) | 3.45 (1.80,6.64) |

^a^ Among women born in ‘Other’ regions, having ‘None/inadequately defined’ antenatal care was extremely rare in one or more groups, leading to non-estimable RRs in Poisson/GEE models in the 20 imputed data sets. We estimated the RR for categories of duration where cell counts were > 0.

**Table S7.** **Sensitivity analysis 2:** The association between duration of residence and preconception health factors (ref = no), stratified by maternal region of birth, compared with women born in Australia. Adjusted relative risks (aRR) and 95% confidence intervals (CI) from robust Poisson^a^ regression models (adjusted for maternal age and year of birth of the child) in a **sensitivity analysis including only women who migrated at ≥18 years of age** (*N* = 184,119).

|  | **Pre-existing diabetes** | **Pre-existing hypertension** | **Pre-existing thyroid condition** | **Smoking^b^** | **Early-pregnancy weight status BMI ≥25 kg/m^2^** |
| --- | --- | --- | --- | --- | --- |
|  | **aRR (95% CI)** | **aRR (95% CI)** | **aRR (95% CI)** | **aRR (95% CI)** | **aRR (95% CI)** |
| ***Duration of residence*** | |  |  |  |  |
| Australia born | 1.00 (reference) | 1.00 (reference) | 1.00 (reference) | 1.00 (reference) | 1.00 (reference) |
| *<2 years* | 0.72 (0.51, 1.03) | 0.47 (0.33, 0.66) | 1.63 (1.49, 1.78) | 0.20 (0.17, 0.23) | 0.74 (0.71, 0.76) |
| *2-5 years* | 1.17 (1.00, 1.37) | 0.49 (0.41, 0.58) | 1.99 (1.90, 2.09) | 0.22 (0.20, 0.23) | 0.78 (0.77, 0.79) |
| *6-10 years* | 1.38 (1.20, 1.59) | 0.59 (0.50, 0.69) | 1.86 (1.77, 1.95) | 0.29 (0.27, 0.31) | 0.84 (0.82, 0.85) |
| *>10 years* | 1.46 (1.22, 1.74) | 0.85 (0.72, 1.01) | 1.46 (1.36, 1.57) | 0.33 (0.30, 0.37) | 0.83 (0.81, 0.85) |
|  |  |  |  |  |  |
| ***Duration of residence stratified by region of birth*** | |  |  |  |  |
| **Australia born** | 1.00 (reference) | 1.00 (reference) | 1.00 (reference) | 1.00 (reference) | 1.00 (reference) |
|  |  |  |  |  |  |
| **New Zealand** | 1.04 (0.68,1.60) | 0.97 (0.69,1.37) | 0.83 (0.69,1.01) | 1.07 (0.97,1.18) | 1.21 (1.17,1.24) |
| *<2 years* | 0.81 (0.11,5.77) | 0.53 (0.08,3.77) | 0.58 (0.22,1.53) | 1.38 (1.06,1.79) | 1.48 (1.37,1.59) |
| *2-5 years* | 1.84 (0.99,3.42) | 0.46 (0.17,1.23) | 0.85 (0.59,1.23) | 1.11 (0.94,1.31) | 1.28 (1.22,1.35) |
| *6-10 years* | 0.98 (0.49,1.96) | 1.42 (0.91,2.22) | 0.82 (0.61,1.10) | 1.01 (0.86,1.19) | 1.18 (1.12,1.24) |
| *>10 years* | 0.40 (0.10,1.59) | 0.92 (0.46,1.83) | 0.87 (0.60,1.25) | 0.98 (0.77,1.25) | 1.08 (1.00,1.17) |
|  |  |  |  |  |  |
| **Oceania** | 2.87 (1.81,4.54) | 0.88^a^ (0.46,1.68) | 0.95 (0.69,1.31) | 0.66 (0.53,0.83) | 1.51 (1.45,1.56) |
| *<2 years* | Non-estimable^a^ | 1.64^a^ (0.41,6.51) | 0.45 (0.11,1.77) | 0.35 (0.16,0.75) | 1.51 (1.37,1.66) |
| *2-5 years* | 2.67^a^ (1.21,5.91) | 0.56^a^ (0.14,2.21) | 0.68 (0.36,1.29) | 0.58 (0.40,0.83) | 1.55 (1.46,1.63) |
| *6-10 years* | 3.80^a^ (1.91,7.55) | 1.43^a^ (0.60,3.41) | 1.38 (0.88,2.15) | 0.74 (0.51,1.09) | 1.48 (1.38,1.58) |
| *>10 years* | 3.55^a^ (1.35,9.34 | Non-estimable^a^ | 0.98 (0.47,2.01) | 1.13 (0.72,1.78) | 1.48 (1.34,1.64) |
|  |  |  |  |  |  |
| **Africa** | 1.40 (1.05,1.87) | 0.93 (0.71,1.22) | 0.99 (0.86,1.13) | 0.18 (0.15,0.22) | 1.06 (1.03,1.09) |
| *<2 years* | 1.01 (0.33,3.12) | 1.51 (0.72,3.15) | 1.02 (0.65,1.60) | 0.12 (0.06,0.24) | 0.87 (0.78,0.97) |
| *2-5 years* | 1.28 (0.73,2.25) | 0.66 (0.36,1.23) | 1.19 (0.95,1.51) | 0.12 (0.08,0.19) | 1.00 (0.94,1.05) |
| *6-10 years* | 1.49 (0.91,2.44) | 0.57 (0.31,1.05) | 0.95 (0.75,1.20) | 0.22 (0.16,0.30) | 1.08 (1.03,1.13) |
| *>10 years* | 1.64 (1.02,2.63) | 1.40 (0.95,2.08) | 0.81 (0.62,1.06) | 0.24 (0.17,0.34) | 1.22 (1.16,1.28) |
|  |  |  |  |  |  |
| **West Asia** | 1.04 (0.69,1.55) | 0.48 (0.30,0.76) | 1.33 (1.16,1.53) | 0.28 (0.24,0.33) | 1.08 (1.04,1.11) |
| *<2 years* | 0.24 (0.03,1.72) | 0.31 (0.08,1.26) | 1.21 (0.85,1.73) | 0.17 (0.11,0.28) | 0.96 (0.89,1.04) |
| *2-5 years* | 0.82 (0.39,1.72) | 0.44 (0.20,0.99) | 1.56 (1.26,1.94) | 0.21 (0.15,0.29) | 1.00 (0.95,1.06) |
| *6-10 years* | 1.69 (0.94,3.05) | 0.28 (0.09,0.88) | 1.25 (0.95,1.63) | 0.36 (0.27,0.50) | 1.10 (1.04,1.17) |
| *>10 years* | 1.18 (0.49,2.83) | 0.95 (0.46,1.98) | 1.12 (0.79,1.59) | 0.55 (0.39,0.79) | 1.37 (1.29,1.46) |
|  |  |  |  |  |  |
| **South Asia** | 1.75 (1.54,1.99) | 0.68 (0.59,0.79) | 2.91 (2.80,3.03) | 0.15 (0.14,0.17) | 0.97 (0.96,0.99) |
| *<2 years* | 0.82 (0.50,1.34) | 0.29 (0.15,0.55) | 2.56 (2.30,2.85) | 0.12 (0.10,0.16) | 0.80 (0.77,0.84) |
| *2-5 years* | 1.62 (1.34,1.97) | 0.49 (0.38,0.64) | 3.04 (2.88,3.22) | 0.11 (0.09,0.13) | 0.91 (0.89,0.93) |
| *6-10 years* | 1.97 (1.64,2.37) | 0.71 (0.56,0.89) | 2.99 (2.82,3.16) | 0.20 (0.17,0.23) | 1.07 (1.05,1.10) |
| *>10 years* | 2.30 (1.74,3.04) | 1.53 (1.18,1.99) | 2.60 (2.35,2.88) | 0.27 (0.22,0.34) | 1.13 (1.09,1.17) |
|  |  |  |  |  |  |
| **South-East/East Asia** | 1.01 (0.86,1.20) | 0.43 (0.36,0.53) | 1.01 (0.94,1.08) | 0.19 (0.18,0.21) | 0.44 (0.42,0.45) |
| *<2 years* | 0.79 (0.43,1.47) | 0.45 (0.23,0.86) | 0.78 (0.61,1.00) | 0.15 (0.11,0.20) | 0.40 (0.36,0.44) |
| *2-5 years* | 0.67 (0.46,0.96) | 0.48 (0.34,0.68) | 1.01 (0.90,1.14) | 0.18 (0.15,0.21) | 0.42 (0.40,0.44) |
| *6-10 years* | 1.12 (0.86,1.46) | 0.37 (0.26,0.53) | 1.04 (0.93,1.16) | 0.20 (0.17,0.23) | 0.44 (0.42,0.46) |
| *>10 years* | 1.43 (1.08,1.88) | 0.47 (0.33,0.67) | 1.04 (0.92,1.18) | 0.24 (0.20,0.28) | 0.48 (0.46,0.51) |
|  |  |  |  |  |  |
| **Europe** | 0.49 (0.33,0.72) | 0.43 (0.31,0.59) | 1.43 (1.31,1.58) | 0.46 (0.41,0.51) | 0.68 (0.66,0.70) |
| *<2 years* | 0.65 (0.16,2.59) | 0.39 (0.10,1.56) | 1.19 (0.80,1.77) | 0.41 (0.28,0.62) | 0.60 (0.52,0.70) |
| *2-5 years* | 0.60 (0.31,1.16) | 0.41 (0.22,0.76) | 1.39 (1.18,1.65) | 0.47 (0.40,0.57) | 0.59 (0.55,0.63) |
| *6-10 years* | 0.29 (0.13,0.65) | 0.44 (0.27,0.73) | 1.46 (1.27,1.67) | 0.49 (0.42,0.57) | 0.74 (0.70,0.78) |
| *>10 years* | 0.78 (0.39,1.56) | 0.50 (0.26,0.97) | 1.46 (1.20,1.78) | 0.42 (0.31,0.55) | 0.79 (0.73,0.85) |
|  |  |  |  |  |  |
| **Other** | 0.49 (0.27,0.88) | 0.62 (0.41,0.94) | 1.57 (1.37,1.79) | 0.36 (0.30,0.43) | 0.75 (0.71,0.79) |
| *<2 years* | Non-estimable^a^ | 0.68 (0.17,2.69) | 1.51 (0.96,2.39) | 0.31 (0.17,0.57) | 0.71 (0.60,0.84) |
| *2-5 years* | 0.13^a^ (0.02,0.94) | 0.33 (0.12,0.87) | 1.49 (1.19,1.87) | 0.37 (0.28,0.49) | 0.71 (0.65,0.77) |
| *6-10 years* | 0.87^a^ (0.41,1.82) | 0.74 (0.40,1.38) | 1.76 (1.45,2.14) | 0.32 (0.23,0.44) | 0.79 (0.73,0.85) |
| *>10 years* | 0.71^a^ (0.23,2.21) | 0.96 (0.46,2.02) | 1.28 (0.92,1.78) | 0.46 (0.31,0.69) | 0.84 (0.75,0.94) |

^a^ Pre-existing diabetes (for women born in Oceania and Other regions) and pre-existing hypertension (for women born in Oceania) were extremely rare in one or more groups, leading to non-estimable RRs in Poisson/GEE models. We estimated the RR for categories of duration where cell counts were > 0

^b^ Having ever smoked (smoker or quit <20 weeks gestation) - non-smoker as the reference.

**Table S8.** **Sensitivity analysis 2:** The association between duration of residence and gestational week of first antenatal care attendance, overall and stratified by maternal region of birth, compared with women born in Australia and first attending antenatal care <12 weeks’ gestation. Adjusted relative risks (aRR) and 95% confidence intervals (CI) from robust Poisson^a^ regression models (adjusted for maternal age and year of birth of the child) ) in a **sensitivity analysis including only women who migrated at ≥18 years of age** (*N* = 184,119).

|  | **Gestational week of first antenatal care attendance**  **(ref= <12 weeks)** | | |
| --- | --- | --- | --- |
|  | **12-17 weeks** | **≥18 weeks** | **None/Inadequately defined** |
|  | **aRR (95% CI)** | **aRR (95% CI)** | **aRR (95% CI)** |
| **Duration of residence** |  |  |  |
| Australia born | 1.00 (reference) | 1.00 (reference) | 1.00 (reference) |
| < 2 years | 1.29 (1.24,1.35) | 2.52 (2.42,2.62) | 1.55 (1.09,2.21) |
| 2-5 years | 1.37 (1.34,1.40) | 1.67 (1.62,1.73) | 0.87 (0.67,1.11) |
| 6-10 years | 1.31 (1.28,1.34) | 1.44 (1.39,1.50) | 0.53 (0.39,0.73) |
| > 10 years | 1.27 (1.23,1.31) | 1.31 (1.24,1.39) | 0.84 (0.59,1.19) |
|  |  |  |  |
| **Duration of residence stratified by region of birth** | |  |  |
| **Australia born** | 1.00 (reference) | 1.00 (reference) | 1.00 (reference) |
|  |  |  |  |
| **New Zealand** | 1.69 (1.62,1.76) | 2.95 (2.81,3.09) | 1.75 (1.11,2.76) |
| *<2 years* | 1.60 (1.29,1.97) | 3.04 (2.55,3.63) | 2.11 (0.30,14.89) |
| *2-5 years* | 1.43 (1.28,1.59) | 2.55 (2.28,2.86) | 2.22 (0.93,5.31) |
| *6-10 years* | 1.35 (1.24,1.48) | 1.77 (1.54,2.02) | 0.84 (0.27,2.60) |
| *>10 years* | 1.30 (1.13,1.48) | 1.78 (1.47,2.15) | 0.52 (0.07,3.68) |
|  |  |  |  |
| **Oceania** | 1.24 (1.19,1.30) | 1.50 (1.40,1.60) | 1.00 (0.65,1.53) |
| *<2 years* | 1.35 (0.92,1.98) | 4.19 (3.59,4.89) | 8.90 (2.37,33.38) |
| *2-5 years* | 1.61 (1.38,1.88) | 2.76 (2.34,3.24) | 3.63 (1.19,11.08) |
| *6-10 years* | 1.32 (1.09,1.61) | 2.86 (2.38,3.44) | Non-estimable^a^ |
| *>10 years* | 1.27 (0.95,1.69) | 2.25 (1.62,3.11) | 4.55 (1.15,17.99) |
|  |  |  |  |
| **Africa** | 1.38 (1.30,1.46) | 2.14 (1.99,2.31) | 1.22 (0.65,2.28) |
| *<2 years* | 1.76 (1.54,1.99) | 3.53 (3.17,3.94) | 2.07 (0.52,8.18) |
| *2-5 years* | 1.60 (1.49,1.73) | 2.72 (2.50,2.97) | 2.57 (1.34,4.92) |
| *6-10 years* | 1.69 (1.58,1.82) | 2.89 (2.65,3.15) | 0.86 (0.28,2.66) |
| *>10 years* | 1.74 (1.62,1.88) | 2.95(2.68,3.25) | 1.71 (0.71,4.11) |
|  |  |  |  |
| **West Asia** | 1.44 (1.29,1.60) | 2.97 (2.69,3.27) | 3.10 (1.48,6.46) |
| *<2 years* | 1.13 (0.98,1.31) | 2.27 (2.00,2.58) | 2.35 (0.98,5.63) |
| *2-5 years* | 1.21 (1.10,1.33) | 1.50 (1.32,1.70) | 0.22 (0.03,1.55) |
| *6-10 years* | 1.18 (1.05,1.32) | 1.47 (1.24,1.73) | 0.32 (0.04,2.26) |
| *>10 years* | 1.22 (1.05,1.41) | 1.65 (1.33,2.05) | 1.14 (0.29,4.52) |
|  |  |  |  |
| **South Asia** | 1.20 (1.13,1.28) | 1.86 (1.72,2.02) | 1.15 (0.63,2.09) |
| *<2 years* | 1.29 (1.22,1.37) | 2.20 (2.07,2.34) | 0.60 (0.27,1.34) |
| *2-5 years* | 1.39 (1.35,1.43) | 1.44 (1.37,1.51) | 0.57 (0.37,0.88) |
| *6-10 years* | 1.38 (1.34,1.43) | 1.24 (1.16,1.32) | 0.27 (0.13,0.54) |
| *>10 years* | 1.37 (1.29,1.45) | 1.04 (0.91,1.18) | 0.74 (0.35,1.57) |
|  |  |  |  |
| **South-East/ East Asia** | 1.24 (1.21,1.27) | 1.52 (1.47,1.58) | 0.84 (0.64,1.10) |
| *<2 years* | 1.24 (1.14,1.35) | 2.70 (2.51,2.90) | 2.59 (1.53,4.38) |
| *2-5 years* | 1.33 (1.28,1.39) | 1.65 (1.55,1.75) | 0.88 (0.55,1.40) |
| *6-10 years* | 1.21 (1.17,1.26) | 1.37 (1.29,1.46) | 0.57 (0.33,0.96) |
| *>10 years* | 1.14 (1.09,1.20) | 0.98 (0.89,1.08) | 0.61 (0.34,1.12) |
|  |  |  |  |
| **Europe** | 1.38 (1.35,1.41) | 1.45 (1.40,1.50) | 0.49 (0.36,0.67) |
| *<2 years* | 1.23 (1.04,1.45) | 2.44 (2.08,2.87) | 2.23 (0.73,6.88) |
| *2-5 years* | 1.31 (1.22,1.40) | 1.60 (1.44,1.77) | 0.71 (0.30,1.71) |
| *6-10 years* | 1.25 (1.17,1.33) | 1.34 (1.20,1.49) | 1.05 (0.56,1.97) |
| *>10 years* | 1.12 (1.01,1.24) | 1.16 (0.97,1.38) | 0.92 (0.34,2.44) |
|  |  |  |  |
| **Other** | 1.19 (1.12,1.26) | 1.69 (1.56,1.82) | 0.77 (0.40,1.49) |
| *<2 years* | 1.17 (0.92,1.47) | 2.85 (2.39,3.40) | Non-estimable^a^ |
| *2-5 years* | 1.31 (1.19,1.45) | 2.18 (1.94,2.44) | 1.83 (0.82,4.08) |
| *6-10 years* | 1.11 (0.99,1.23) | 1.40 (1.19,1.64) | 0.77 (0.25,2.38) |
| *>10 years* | 1.21 (1.04,1.41) | 1.37 (1.07,1.77) | 1.16 (0.29,4.65) |

^a^ Among women born in ‘Other’ regions, having ‘None/inadequately defined’ antenatal care was extremely rare in one or more groups, leading to non-estimable RRs in Poisson/GEE models. We estimated the RR for categories of duration where cell counts were > 0.

**Table S9**. **Sensitivity analysis 2:** The association between duration of residence and pregnancy and birth outcomes, overall and stratified by maternal region of birth (ref = no), compared with women born in Australia. Adjusted relative risks (aRR) and 95% confidence intervals (CI) from robust Poisson^a^ regression models (adjusted for maternal age and year of birth of the child) in a **sensitivity analysis including only women who migrated at ≥18 years of age** (*N* = 184,119).

|  | **Stillbirth** | **Preeclampsia** | **Preterm birth** | **Gestational diabetes mellitus** |
| --- | --- | --- | --- | --- |
|  | **aRR (95% CI)** | **aRR (95% CI)** | **aRR (95% CI)** | **aRR (95% CI)** |
| ***Duration of residence stratified*** |  |  |  |  |
| Australia born | 1.00 (reference) | 1.00 (reference) | 1.00 (reference) | 1.00 (reference) |
| < 2 years | 1.36 (0.92, 2.00) | 0.75 (0.62, 0.91) | 0.83 (0.74, 0.92) | 1.88 (1.77, 1.99) |
| 2-5 years | 0.98 (0.76, 1.27) | 0.68 (0.60, 0.76) | 0.88 (0.83, 0.94) | 2.16 (2.09, 2.23) |
| 6-10 years | 1.20 (0.95, 1.53) | 0.64 (0.57, 0.73) | 0.93 (0.87, 0.98) | 2.13 (2.06, 2.19) |
| > 10 years | 1.29 (0.95, 1.77) | 0.82 (0.70, 0.96) | 0.90 (0.83, 0.98) | 2.07 (1.99, 2.16) |
|  |  |  |  |  |
| ***Duration of residence stratified by region of birth*** |  |  |  |  |
| **Australia** | 1.00 (reference) | 1.00 (reference) | 1.00 (reference) | 1.00 (reference) |
|  |  |  |  |  |
| **New Zealand** | 1.27 (0.68,2.38) | 1.12 (0.86,1.44) | 0.85 (0.72,1.01) | 1.17 (1.05,1.31) |
| <2 years | 1.67 (0.23,11.93) | 1.15 (0.49,2.73) | 0.72 (0.37,1.42) | 1.46 (0.99,2.15) |
| 2-5 years | 0.88 (0.22,3.53) | 1.12 (0.71,1.77) | 1.04 (0.78,1.38) | 1.23 (1.00,1.52) |
| 6-10 years | 1.62 (0.67,3.91) | 1.01 (0.66,1.54) | 0.77 (0.58,1.02) | 1.07 (0.90,1.29) |
| >10 years | 1.12 (0.28,4.48) | 1.37 (0.82,2.31) | 0.83 (0.58,1.20) | 1.22 (0.98,1.51) |
|  |  |  |  |  |
| **Oceania** | 0.79 (0.20,3.17) | 1.41 (0.95,2.09) | 0.95 (0.71,1.26) | 2.57 (2.27,2.90) |
| <2 years | Non-estimable^a^ | 1.60 (0.61,4.21) | 0.77 (0.33,1.80) | 2.28 (1.55,3.33) |
| 2-5 years | Non-estimable^a^ | 0.89 (0.40,1.96) | 0.77 (0.46,1.29) | 2.30 (1.85,2.86) |
| 6-10 years | 1.27 (0.18,9.01) | 1.50 (0.76,2.97) | 1.11 (0.71,1.76) | 2.74 (2.24,3.35) |
| >10 years | 2.41 (0.34,17.02) | 2.50 (1.14,5.48) | 1.19 (0.64,2.24) | 2.98 (2.31,3.85) |
|  |  |  |  |  |
| **Africa** | 2.21 (1.52,3.21) | 0.92 (0.74,1.15) | 0.86 (0.75,0.98) | 1.47 (1.36,1.58) |
| <2 years | 2.94 (1.11,7.83) | 1.23 (0.70,2.15) | 0.78 (0.51,1.20) | 1.18 (0.89,1.56) |
| 2-5 years | 1.82 (0.86,3.82) | 0.85 (0.56,1.27) | 0.65 (0.49,0.86) | 1.35 (1.16,1.56) |
| 6-10 years | 1.97 (0.98,3.96) | 0.75 (0.49,1.17) | 0.88 (0.70,1.11) | 1.38 (1.20,1.58) |
| >10 years | 2.68 (1.43,5.02) | 1.11 (0.75,1.65) | 1.11 (0.89,1.38) | 1.80 (1.60,2.03) |
|  |  |  |  |  |
| **West Asia** | 0.52 (0.21,1.25) | 0.45 (0.32,0.65) | 0.74 (0.63,0.88) | 1.86 (1.72,2.01) |
| <2 years | Non-estimable^a^ | 0.22 (0.07,0.67) | 0.83 (0.59,1.18) | 1.56 (1.27,1.91) |
| 2-5 years | 1.06^a^ (0.40,2.84) | 0.49 (0.28,0.84) | 0.77 (0.59,1.00) | 1.91 (1.68,2.16) |
| 6-10 years | Non-estimable^a^ | 0.65 (0.36,1.17) | 0.55 (0.37,0.80) | 1.87 (1.61,2.16) |
| >10 years | 0.65 (0.09,4.63) | 0.34 (0.11,1.04) | 0.88 (0.60,1.30) | 2.01 (1.69,2.38) |
|  |  |  |  |  |
| **South Asia** | 1.38 (1.11,1.72) | 0.78 (0.70,0.86) | 1.08 (1.02,1.13) | 2.84 (2.77,2.92) |
| <2 years | 1.76 (1.07,2.91) | 0.89 (0.69,1.15) | 1.05 (0.91,1.21) | 2.32 (2.16,2.50) |
| 2-5 years | 0.98 (0.68,1.43) | 0.76 (0.65,0.89) | 0.97 (0.89,1.05) | 2.82 (2.72,2.93) |
| 6-10 years | 1.64 (1.19,2.26) | 0.70 (0.58,0.85) | 1.18 (1.08,1.28) | 2.96 (2.85,3.08) |
| >10 years | 1.74 (1.02,2.98) | 0.93 (0.69,1.26) | 1.21 (1.05,1.40) | 3.02 (2.84,3.20) |
|  |  |  |  |  |
| **South-East/East Asia** | 0.86 (0.64,1.16) | 0.54 (0.47,0.62) | 0.79 (0.73,0.84) | 2.02 (1.96,2.09) |
| <2 years | 0.75 (0.28,2.00) | 0.48 (0.31,0.77) | 0.64 (0.51,0.82) | 1.80 (1.61,2.00) |
| 2-5 years | 0.97 (0.60,1.57) | 0.48 (0.37,0.62) | 0.85 (0.76,0.95) | 2.03 (1.91,2.14) |
| 6-10 years | 0.87 (0.54,1.42) | 0.50 (0.39,0.64) | 0.79 (0.70,0.88) | 2.08 (1.98,2.19) |
| >10 years | 0.80 (0.44,1.46) | 0.75 (0.58,0.96) | 0.77 (0.67,0.88) | 2.05 (1.93,2.18) |
|  |  |  |  |  |
| **Europe** | 1.01 (0.64,1.60) | 0.52 (0.40,0.66) | 0.67 (0.59,0.76) | 0.91 (0.84,0.99) |
| <2 years | 1.56 (0.39,6.25) | 0.37 (0.12,1.13) | 0.30 (0.14,0.62) | 1.04 (0.77,1.41) |
| 2-5 years | 1.02 (0.45,2.27) | 0.51 (0.33,0.79) | 0.72 (0.58,0.90) | 0.82 (0.71,0.96) |
| 6-10 years | 0.78 (0.35,1.76) | 0.55 (0.37,0.80) | 0.73 (0.60,0.88) | 0.95 (0.84,1.08) |
| >10 years | 1.38 (0.57,3.34) | 0.60 (0.34,1.06) | 0.62 (0.46,0.84) | 0.98 (0.83,1.16) |
|  |  |  |  |  |
| **Other** | 0.59 (0.25,1.43) | 0.75 (0.55,1.02) | 0.79 (0.66,0.94) | 0.94 (0.84,1.06) |
| <2 years | 1.35 (0.19,9.58) | 1.00 (0.42,2.39) | 0.29 (0.11,0.76) | 0.74 (0.46,1.18) |
| 2-5 years | 0.34 (0.05,2.39) | 0.80 (0.49,1.30) | 0.85 (0.64,1.11) | 0.96 (0.79,1.17) |
| 6-10 years | 0.66 (0.16,2.64) | 0.69 (0.40,1.18) | 0.86 (0.65,1.13) | 0.97 (0.80,1.17) |
| >10 years | 0.67 (0.09,4.80) | 0.72 (0.33,1.61) | 0.82 (0.55,1.24) | 1.02 (0.79,1.32) |

^a^ For women born in Oceania and West Asia, stillbirths were extremely rare in one or more groups, leading to non-estimable RRs in Poisson/GEE models.

We estimated the RR for categories of duration where cell counts were > 0.

**Table S10.** **Sensitivity analysis 3:** he association between duration of residence and preconception health factors (ref = no), stratified by maternal region of birth, compared with women born in Australia. Adjusted relative risks (aRR) and 95% confidence intervals (CI) from robust Poisson^a^ regression models (adjusted for maternal age and year of birth of the child) from a **sensitivity analysis using imputed data (50 imputations) for missing duration of residence, smoking, and OWOB** (*N* =217,136).

|  | **Pre-existing diabetes** | | | **Pre-existing hypertension** | | **Pre-existing thyroid condition** | | **Smoking^b^** | | **Early-pregnancy weight status BMI ≥25 kg/m^2^** | |
| --- | --- | --- | --- | --- | --- | --- | --- | --- | --- | --- | --- |
|  | **aRR** | **95% CI** | | **aRR** | **95% CI** | **aRR** | **95% CI** | **aRR** | **95% CI** | **aRR** | **95% CI** |
| ***Duration of residence*** | |  | |  |  |  |  |  |  |  |  |
| Australia born | 1.00 (reference) |  | | 1.00 (reference) |  | 1.00 (reference) |  | 1.00 (reference) |  | 1.00 (reference) |  |
| *<2 years* | 0.83 | (0.59, 1.15) | | 0.50 | (0.36, 0.69) | 1.60 | (1.46, 1.75) | 0.21 | (0.18, 0.23) | 0.75 | (0.73, 0.77) |
| *2-5 years* | 1.20 | (1.03, 1.39) | | 0.49 | (0.41, 0.58) | 1.98 | (1.89, 2.07) | 0.20 | (0.19, 0.22) | 0.78 | (0.77, 0.80) |
| *6-10 years* | 1.33 | (1.17, 1.52) | | 0.58 | (0.49, 0.67) | 1.81 | (1.72, 1.89) | 0.28 | (0.26, 0.30) | 0.83 | (0.82, 0.85) |
| *>10 years* | 1.27 | (1.12, 1.45) | | 0.76 | (0.68, 0.86) | 1.28 | (1.21, 1.35) | 0.50 | (0.48, 0.53) | 0.86 | (0.84, 0.87) |
|  |  |  | |  |  |  |  |  |  |  |  |
| ***Duration of residence stratified by region of birth*** | | | | |  |  |  |  |  |  |  |
| **Australia born** | 1.00 (reference) |  | | 1.00 (reference) |  | 1.00 (reference) |  | 1.00 (reference) |  | 1.00 (reference) |  |
|  |  |  | |  |  |  |  |  |  |  |  |
| **New Zealand** |  |  | |  |  |  |  | 1.16 | (1.09, 1.23) | 1.24 | (1.22, 1.27) |
| *<2 years* | 0.89 | (0.14, 5.69) | | 0.60 | (0.10, 3.64) | 0.53 | (0.21, 1.32) | 1.26 | (0.97, 1.62) | 1.36 | (1.24, 1.48) |
| *2-5 years* | 1.70 | (0.93, 3.12) | | 0.50 | (0.20, 1.26) | 0.82 | (0.58, 1.15) | 1.00 | (0.85, 1.17) | 1.24 | (1.18, 1.31) |
| *6-10 years* | 1.02 | (0.54, 1.93) | | 1.27 | (0.83, 1.94) | 0.73 | (0.55, 0.98) | 0.96 | (0.84, 1.10) | 1.19 | (1.14, 1.25) |
| *>10 years* | 1.46 | (0.99, 2.16) | | 1.27 | (0.93, 1.75) | 0.80 | (0.65, 0.98) | 1.32 | (1.21, 1.43) | 1.25 | (1.21, 1.29) |
|  |  |  | |  |  |  |  |  |  |  |  |
| **Oceania** |  |  | |  |  |  |  | 0.79 | (0.68, 0.91) | 1.48 | (1.44, 1.52) |
| *<2 years* | Non-estimable^a^ | | | 1.49 | (0.38, 5.93) | 0.50 | (0.13, 1.92) | 0.38 | (0.18, 0.81) | 1.51 | (1.37, 1.66) |
| *2-5 years* | 2.46^a^ | (1.12, 5.40) | | 0.52 | (0.13, 2.02) | 0.75 | (0.41, 1.36) | 0.52 | (0.37, 0.74) | 1.53 | (1.45, 1.61) |
| *6-10 years* | 3.09^a^ | (1.57, 6.07) | | 1.14 | (0.48, 2.71) | 1.28 | (0.84, 1.95) | 0.67 | (0.49, 0.91) | 1.45 | (1.37, 1.54) |
| *>10 years* | 2.89^a^ | (1.70, 4.90) | | Non-estimable^a^ | | 0.79 | (0.52, 1.20) | 1.14 | (0.95, 1.38) | 1.45 | (1.39, 1.52) |
|  |  |  | |  |  |  |  |  |  |  |  |
|  |  |  | |  |  |  |  |  |  |  |  |
| **Africa** |  |  | |  |  |  |  | 0.26 | (0.24, 0.30) | 1.02 | (0.99, 1.04) |
| *<2 years* | 0.99 | (0.34, 2.94) | | 1.28 | (0.62, 2.66) | 0.98 | (0.63, 1.56) | 0.12 | (0.06, 0.24) | 0.89 | (0.80, 0.98) |
| *2-5 years* | 1.28 | (0.75, 2.18) | | 0.64 | (0.34, 1.19) | 1.15 | (0.92, 1.44) | 0.13 | (0.09, 0.19) | 0.96 | (0.91, 1.02) |
| *6-10 years* | 1.33 | (0.84, 2.11) | | 0.62 | (0.36, 1.06) | 0.94 | (0.76, 1.17) | 0.20 | (0.15, 0.26) | 1.02 | (0.98, 1.07) |
| *>10 years* | 1.20 | (0.84, 1.70) | | 1.04 | (0.77, 1.39) | 0.87 | (0.73, 1.02) | 0.40 | (0.34, 0.46) | 1.05 | (1.02, 1.09) |
|  |  |  | |  |  |  |  |  |  |  |  |
| **West Asia** |  |  | |  |  |  |  | 0.33 | (0.29, 0.37) | 1.08 | (1.05, 1.10) |
| *<2 years* | 0.37 | (0.07, 2.04) | | 0.40 | (0.11, 1.48) | 1.22 | (0.87, 1.72) | 0.18 | (0.12, 0.29) | 0.98 | (0.91, 1.06) |
| *2-5 years* | 0.83 | (0.40, 1.69) | | 0.56 | (0.28, 1.11) | 1.53 | (1.25, 1.88) | 0.19 | (0.14, 0.26) | 0.99 | (0.94, 1.04) |
| *6-10 years* | 1.47 | (0.83, 2.60) | | 0.46 | (0.19, 1.09) | 1.27 | (1.00, 1.62) | 0.31 | (0.23, 0.41) | 1.07 | (1.01, 1.13) |
| *>10 years* | 1.15 | (0.67, 1.98) | | 0.81 | (0.50, 1.33) | 1.42 | (1.18, 1.71) | 0.56 | (0.47, 0.68) | 1.18 | (1.13, 1.23) |
|  |  |  | |  |  |  |  |  |  |  |  |
| **South Asia** |  |  | |  |  |  |  | 0.15 | (0.13, 0.16) | 0.97 | (0.96, 0.98) |
| *<2 years* | 0.92 | (0.57, 1.49) | | 0.32 | (0.17, 0.60) | 2.59 | (2.33, 2.88) | 0.12 | (0.09, 0.15) | 0.83 | (0.79, 0.86) |
| *2-5 years* | 1.61 | (1.34, 1.94) | | 0.49 | (0.38, 0.63) | 3.03 | (2.87, 3.19) | 0.10 | (0.08, 0.11) | 0.91 | (0.89, 0.93) |
| *6-10 years* | 1.93 | (1.62, 2.30) | | 0.68 | (0.54, 0.85) | 2.99 | (2.83, 3.16) | 0.17 | (0.15, 0.20) | 1.06 | (1.04, 1.08) |
| *>10 years* | 2.10 | (1.68, 2.63) | | 1.15 | (0.91, 1.44) | 2.45 | (2.25, 2.66) | 0.25 | (0.21, 0.29) | 1.04 | (1.01, 1.07) |
|  |  |  | |  |  |  |  |  |  |  |  |
| **South-East/East Asia** |  |  | |  |  |  |  | 0.22 | (0.20, 0.23) | 0.47 | (0.46, 0.48) |
| *<2 years* | 0.84 | (0.45, 1.56) | | 0.48 | (0.26, 0.87) | 0.79 | (0.62, 1.02) | 0.15 | (0.11, 0.20) | 0.41 | (0.37, 0.45) |
| *2-5 years* | 0.75 | (0.53, 1.06) | | 0.47 | (0.34, 0.65) | 1.01 | (0.90, 1.14) | 0.17 | (0.14, 0.20) | 0.43 | (0.41, 0.45) |
| *6-10 years* | 1.07 | (0.83, 1.39) | | 0.36 | (0.25, 0.50) | 1.03 | (0.93, 1.14) | 0.18 | (0.16, 0.21) | 0.44 | (0.42, 0.46) |
| *>10 years* | 1.19 | (0.98, 1.46) | | 0.58 | (0.46, 0.72) | 0.96 | (0.88, 1.05) | 0.30 | (0.27, 0.33) | 0.53 | (0.51, 0.55) |
|  |  |  |  |  |  |  |  |  |  |  |  |
| **Europe** |  |  | |  |  |  |  | 0.55 | (0.52, 0.60) | 0.73 | (0.71, 0.75) |
| *<2 years* | 0.78 | (0.22, 2.84) | | 0.38 | (0.10, 1.48) | 1.24 | (0.86, 1.78) | 0.41 | (0.28, 0.62) | 0.63 | (0.55, 0.72) |
| *2-5 years* | 0.67 | (0.35, 1.26) | | 0.38 | (0.21, 0.70) | 1.36 | (1.16, 1.60) | 0.47 | (0.40, 0.55) | 0.60 | (0.56, 0.64) |
| *6-10 years* | 0.44 | (0.23, 0.85) | | 0.42 | (0.26, 0.69) | 1.43 | (1.25, 1.63) | 0.47 | (0.40, 0.55) | 0.73 | (0.70, 0.77) |
| *>10 years* | 0.82 | (0.57, 1.18) | | 0.54 | (0.38, 0.75) | 1.27 | (1.13, 1.42) | 0.69 | (0.63, 0.76) | 0.84 | (0.81, 0.87) |
|  |  |  | |  |  |  |  |  |  |  |  |
| **Other** |  |  | |  |  |  |  | 0.44 | (0.39, 0.50) | 0.82 | (0.79, 0.84) |
| *<2 years* | Non-estimable^a^ | | | 0.61 | (0.15, 2.39) | 1.35 | (0.86, 2.09) | 0.34 | (0.19, 0.59) | 0.74 | (0.63, 0.87) |
| *2-5 years* | 0.16^a^ | (0.02, 1.01) | | 0.40 | (0.16, 0.99) | 1.44 | (1.15, 1.79) | 0.35 | (0.27, 0.46) | 0.72 | (0.66, 0.77) |
| *6-10 years* | 0.75^a^ | (0.37, 1.55) | | 0.74 | (0.41, 1.33) | 1.65 | (1.36, 1.99) | 0.31 | (0.23, 0.42) | 0.79 | (0.74, 0.85) |
| *>10 years* | 0.65^a^ | (0.33, 1.27) | | 0.76 | (0.46, 1.24) | 1.16 | (0.95, 1.41) | 0.65 | (0.55, 0.77) | 0.95 | (0.90, 1.00) |

^a^ Pre-existing diabetes (for women born in Oceania and Other regions) was extremely rare in one or more groups, leading to non-estimable RRs in Poisson/GEE models in the 20 imputed data sets. We estimated the RR for categories of duration where cell counts were > 0.

^b^ Having ever smoked (smoker or quit <20 weeks gestation) - non-smoker as the reference

**Table S11. Sensitivity analysis 3:** The association between duration of residence and gestational week of first antenatal care attendance, overall and stratified by maternal region of birth, compared with women born in Australia and first attending antenatal care <12 weeks’ gestation. Adjusted relative risks (aRR) and 95% confidence intervals (CI) from robust Poisson^a^ regression models (adjusted for maternal age and year of birth of the child) from a **sensitivity analysis using imputed data (50 imputations) for missing duration of residence, smoking, and OWOB** (*N* =217,136).

|  | **Gestational week of first antenatal care attendance**  **(ref= <12 weeks)** | | | | | |
| --- | --- | --- | --- | --- | --- | --- |
|  | **12-17 weeks** | | **≥18 weeks** | | **None/Inadequately defined** | |
|  | **aRR** | **95% CI** | **aRR** | **95% CI** | **aRR** | **95% CI** |
| **Duration of residence** |  |  |  |  |  |  |
| Australia born | 1.00 (reference) |  | 1.00 (reference) |  | 1.00 (reference) |  |
| < 2 years | 1.36 | (1.31, 1.41) | 2.50 | (2.41, 2.60) | 1.84 | (1.41, 2.41) |
| 2-5 years | 1.43 | (1.40, 1.46) | 1.78 | (1.73, 1.84) | 1.06 | (0.87, 1.29) |
| 6-10 years | 1.37 | (1.34, 1.40) | 1.63 | (1.57, 1.68) | 0.89 | (0.72, 1.10) |
| > 10 years | 1.29 | (1.27, 1.32) | 1.50 | (1.45, 1.55) | 1.15 | (0.96, 1.38) |
|  |  |  |  |  |  |  |
| **Duration of residence stratified by region of birth** | | |  |  |  |  |
| **Australia born** | 1.00 (reference) |  | 1.00 (reference) |  | 1.00 (reference) |  |
|  |  |  |  |  |  |  |
| **New Zealand** |  |  |  |  |  |  |
| *<2 years* | 1.63 | (1.35, 1.98) | 2.93 | (2.47, 3.48) | 3.52 | (1.03, 12.01) |
| *2-5 years* | 1.50 | (1.36, 1.65) | 2.58 | (2.33, 2.85) | 2.90 | (1.55, 5.41) |
| *6-10 years* | 1.40 | (1.29, 1.52) | 1.98 | (1.78, 2.20) | 1.56 | (0.75, 3.26) |
| *>10 years* | 1.34 | (1.25, 1.43) | 2.05 | (1.90, 2.21) | 1.64 | (0.98, 2.74) |
|  |  |  |  |  |  |  |
| **Oceania** |  |  |  |  |  |  |
| *<2 years* | 1.40 | (0.98, 2.01) | 3.99 | (3.39, 4.69) | 6.89 | (1.93, 24.62) |
| *2-5 years* | 1.57 | (1.34, 1.83) | 2.64 | (2.27, 3.08) | 4.27 | (1.91, 9.57) |
| *6-10 years* | 1.41 | (1.20, 1.66) | 2.72 | (2.34, 3.16) | 3.27 | (1.25, 8.52) |
| *>10 years* | 1.31 | (1.15, 1.49) | 2.26 | (1.96, 2.60) | 3.75 | (2.01, 7.02) |
|  |  |  |  |  |  |  |
| **Africa** |  |  |  |  |  |  |
| *<2 years* | 1.85 | (1.64, 2.07) | 3.61 | (3.27, 3.99) | 4.00 | (1.76, 9.07) |
| *2-5 years* | 1.69 | (1.58, 1.81) | 2.87 | (2.66, 3.10) | 2.48 | (1.43, 4.31) |
| *6-10 years* | 1.74 | (1.64, 1.85) | 2.96 | (2.75, 3.18) | 1.78 | (0.95, 3.34) |
| *>10 years* | 1.64 | (1.57, 1.72) | 2.65 | (2.50, 2.80) | 2.11 | (1.41, 3.16) |
|  |  |  |  |  |  |  |
| **West Asia** |  |  |  |  |  |  |
| *<2 years* | 1.19 | (1.03, 1.36) | 2.30 | (2.04, 2.60) | 2.13 | (0.98, 4.63) |
| *2-5 years* | 1.28 | (1.17, 1.39) | 1.74 | (1.56, 1.94) | 1.02 | (0.46, 2.25) |
| *6-10 years* | 1.26 | (1.14, 1.38) | 1.78 | (1.56, 2.03) | 0.90 | (0.30, 2.73) |
| *>10 years* | 1.26 | (1.17, 1.37) | 1.73 | (1.54, 1.94) | 1.53 | (0.84, 2.78) |
|  |  |  |  |  |  |  |
| **South Asia** |  |  |  |  |  |  |
| *<2 years* | 1.36 | (1.29, 1.44) | 2.20 | (2.07, 2.34) | 0.85 | (0.48, 1.53) |
| *2-5 years* | 1.47 | (1.43, 1.51) | 1.57 | (1.50, 1.64) | 0.62 | (0.43, 0.90) |
| *6-10 years* | 1.45 | (1.40, 1.49) | 1.39 | (1.31, 1.47) | 0.47 | (0.28, 0.77) |
| *>10 years* | 1.36 | (1.30, 1.42) | 1.26 | (1.16, 1.37) | 0.75 | (0.46, 1.22) |
|  |  |  |  |  |  |  |
| **South-East/ East Asia** |  |  |  |  |  |  |
| *<2 years* | 1.29 | (1.19, 1.39) | 2.62 | (2.45, 2.81) | 2.25 | (1.40, 3.61) |
| *2-5 years* | 1.36 | (1.31, 1.41) | 1.72 | (1.63, 1.82) | 1.11 | (0.77, 1.59) |
| *6-10 years* | 1.27 | (1.22, 1.31) | 1.48 | (1.40, 1.57) | 0.81 | (0.56, 1.18) |
| *>10 years* | 1.20 | (1.16, 1.24) | 1.16 | (1.09, 1.23) | 0.92 | (0.68, 1.25) |
|  |  |  |  |  |  |  |
| **Europe** |  |  |  |  |  |  |
| *<2 years* | 1.38 | (1.19, 1.59) | 2.48 | (2.13, 2.88) | 2.14 | (0.85, 5.43) |
| *2-5 years* | 1.36 | (1.28, 1.45) | 1.70 | (1.54, 1.88) | 0.85 | (0.43, 1.71) |
| *6-10 years* | 1.31 | (1.24, 1.39) | 1.49 | (1.36, 1.64) | 1.08 | (0.64, 1.82) |
| *>10 years* | 1.21 | (1.15, 1.27) | 1.17 | (1.07, 1.28) | 0.68 | (0.41, 1.14) |
|  |  |  |  |  |  |  |
| **Other** |  |  |  |  |  |  |
| *<2 years* | 1.18 | (0.95, 1.47) | 2.71 | (2.28, 3.24) | 3.01 | (1.06, 8.59) |
| *2-5 years* | 1.27 | (1.16, 1.40) | 2.10 | (1.88, 2.34) | 1.75 | (0.87, 3.51) |
| *6-10 years* | 1.14 | (1.03, 1.26) | 1.54 | (1.33, 1.77) | 1.21 | (0.54, 2.72) |
| *>10 years* | 1.28 | (1.18, 1.38) | 1.39 | (1.22, 1.59) | 1.61 | (0.88, 2.95) |

* Among women born in ‘Other’ regions, having ‘None/inadequately defined’ antenatal care was extremely rare in one or more groups, leading to non-estimable RRs in Poisson/GEE models in the 20 imputed data sets. We estimated the RR for categories of duration where cell counts were > 0.

**Table S12**. **Sensitivity analysis 3:** The association between duration of residence and pregnancy and birth outcomes, overall and stratified by maternal region of birth, compared with women born in Australia. Adjusted relative risks (aRR) and 95% confidence intervals (CI) from robust Poisson^a^ regression models (adjusted for maternal age and year of birth of the child) from a **sensitivity analysis using imputed data (50 imputations) for missing duration of residence, smoking, and OWOB** (*N* =217,136).

|  | **Stillbirth** | | **Preeclampsia** | | **Preterm birth** | | **Gestational diabetes mellitus** | |
| --- | --- | --- | --- | --- | --- | --- | --- | --- |
|  | **aRR** | **95% CI** | **aRR** | **95% CI** | **aRR** | **95% CI** | **aRR** | **95% CI** |
| ***Duration of residence stratified*** |  |  |  |  |  |  |  |  |
| Australia born | 1.00 (reference) |  | 1.00 (reference) |  | 1.00 (reference) |  | 1.00 (reference) |  |
| < 2 years | 1.48 | (1.03, 2.13) | 0.77 | (0.64, 0.92) | 0.90 | (0.81, 0.99) | 1.85 | (1.75, 1.95) |
| 2-5 years | 1.11 | (0.88, 1.40) | 0.71 | (0.64, 0.79) | 0.92 | (0.87, 0.97) | 2.13 | (2.07, 2.20) |
| 6-10 years | 1.27 | (1.03, 1.58) | 0.69 | (0.61, 0.76) | 0.94 | (0.89, 1.00) | 2.05 | (1.99, 2.11) |
| > 10 years | 1.27 | (1.03, 1.55) | 0.84 | (0.77, 0.93) | 0.93 | (0.88, 0.98) | 1.83 | (1.78, 1.89) |
|  |  |  |  |  |  |  |  |  |
| ***Duration of residence stratified by region of birth*** | |  |  |  |  |  |  |  |
| **New Zealand** |  |  |  |  |  |  |  |  |
| <2 years | 1.22 | (0.17, 8.73) | 1.03 | (0.46, 2.35) | 0.83 | (0.46, 1.50) | 1.25 | (0.87, 1.81) |
| 2-5 years | 0.70 | (0.18, 2.75) | 1.07 | (0.70, 1.65) | 1.08 | (0.84, 1.39) | 1.16 | (0.95, 1.42) |
| 6-10 years | 1.31 | (0.59, 2.92) | 0.82 | (0.55, 1.24) | 0.76 | (0.59, 0.98) | 1.04 | (0.88, 1.23) |
| >10 years | 1.47 | (0.83, 2.59) | 1.31 | (1.03, 1.67) | 0.90 | (0.76, 1.06) | 1.30 | (1.17, 1.46) |
|  |  |  |  |  |  |  |  |  |
| **Oceania** |  |  |  |  |  |  |  |  |
| <2 years | Non-estimable^a^ | | 1.61 | (0.62, 4.19) | 0.81 | (0.36, 1.84) | 2.24 | (1.54, 3.24) |
| 2-5 years | Non-estimable^a^ | | 0.95 | (0.45, 2.02) | 0.82 | (0.50, 1.32) | 2.25 | (1.83, 2.78) |
| 6-10 years | 2.00^a^ | (0.59, 6.76) | 1.65 | (0.97, 2.81) | 1.10 | (0.74, 1.63) | 2.52 | (2.09, 3.05) |
| >10 years | 2.78^a^ | (1.24, 6.27) | 1.95 | (1.31, 2.90) | 0.96 | (0.69, 1.32) | 2.43 | (2.10, 2.82) |
|  |  |  |  |  |  |  |  |  |
| **Africa** |  |  |  |  |  |  |  |  |
| <2 years | 3.13 | (1.28, 7.66) | 1.09 | (0.63, 1.90) | 0.99 | (0.70, 1.41) | 1.19 | (0.91, 1.55) |
| 2-5 years | 1.94 | (0.99, 3.81) | 0.85 | (0.58, 1.24) | 0.72 | (0.56, 0.92) | 1.34 | (1.17, 1.55) |
| 6-10 years | 1.86 | (1.00, 3.45) | 0.73 | (0.50, 1.07) | 0.92 | (0.75, 1.11) | 1.32 | (1.16, 1.50) |
| >10 years | 2.59 | (1.77, 3.79) | 1.00 | (0.79, 1.27) | 1.11 | (0.98, 1.26) | 1.56 | (1.43, 1.69) |
|  |  |  |  |  |  |  |  |  |
| **West Asia** |  |  |  |  |  |  |  |  |
| <2 years | Non-estimable^a^ | | 0.33 | (0.13, 0.84) | 0.89 | (0.65, 1.24) | 1.67 | (1.37, 2.02) |
| 2-5 years | 1.20^a^ | (0.54, 2.65) | 0.54 | (0.33, 0.88) | 0.85 | (0.67, 1.07) | 1.87 | (1.66, 2.11) |
| 6-10 years | 0.06^a^ | (0.02, 0.25) | 0.73 | (0.45, 1.20) | 0.69 | (0.51, 0.94) | 1.84 | (1.60, 2.10) |
| >10 years | 1.56^a^ | (0.80, 3.03) | 0.58 | (0.36, 0.94) | 0.87 | (0.70, 1.08) | 1.98 | (1.78, 2.20) |
|  |  |  |  |  |  |  |  |  |
| **South Asia** |  |  |  |  |  |  |  |  |
| <2 years | 1.89 | (1.18, 3.03) | 0.88 | (0.69, 1.13) | 1.06 | (0.92, 1.21) | 2.37 | (2.20, 2.55) |
| 2-5 years | 1.19 | (0.86, 1.65) | 0.78 | (0.67, 0.90) | 0.98 | (0.91, 1.06) | 2.82 | (2.72, 2.92) |
| 6-10 years | 1.64 | (1.21, 2.23) | 0.74 | (0.62, 0.87) | 1.15 | (1.06, 1.25) | 2.95 | (2.84, 3.06) |
| >10 years | 1.43 | (0.91, 2.26) | 0.84 | (0.67, 1.05) | 1.16 | (1.04, 1.30) | 2.79 | (2.66, 2.93) |
|  |  |  |  |  |  |  |  |  |
| **South-East/East Asia** |  |  |  |  |  |  |  |  |
| <2 years | 0.90 | (0.37, 2.16) | 0.54 | (0.35, 0.83) | 0.76 | (0.61, 0.95) | 1.76 | (1.59, 1.96) |
| 2-5 years | 0.96 | (0.61, 1.50) | 0.54 | (0.42, 0.68) | 0.89 | (0.80, 0.99) | 1.97 | (1.87, 2.08) |
| 6-10 years | 0.99 | (0.66, 1.50) | 0.58 | (0.47, 0.72) | 0.85 | (0.76, 0.94) | 1.99 | (1.89, 2.09) |
| >10 years | 0.75 | (0.50, 1.13) | 0.72 | (0.61, 0.86) | 0.85 | (0.78, 0.93) | 1.98 | (1.90, 2.06) |
|  |  |  |  |  |  |  |  |  |
| **Europe** |  |  |  |  |  |  |  |  |
| <2 years | 1.48 | (0.41, 5.36) | 0.54 | (0.21, 1.39) | 0.51 | (0.29, 0.89) | 0.91 | (0.68, 1.22) |
| 2-5 years | 1.04 | (0.49, 2.23) | 0.58 | (0.38, 0.88) | 0.75 | (0.61, 0.92) | 0.80 | (0.69, 0.94) |
| 6-10 years | 0.86 | (0.41, 1.81) | 0.63 | (0.44, 0.89) | 0.74 | (0.62, 0.89) | 0.90 | (0.80, 1.02) |
| >10 years | 1.04 | (0.62, 1.74) | 0.78 | (0.61, 1.00) | 0.80 | (0.70, 0.91) | 1.04 | (0.95, 1.14) |
|  |  |  |  |  |  |  |  |  |
| **Other** |  |  |  |  |  |  |  |  |
| <2 years | 1.30 | (0.19, 8.80) | 0.88 | (0.37, 2.06) | 0.56 | (0.27, 1.16) | 0.73 | (0.46, 1.17) |
| 2-5 years | 0.78 | (0.21, 2.81) | 0.75 | (0.46, 1.20) | 0.98 | (0.77, 1.24) | 0.94 | (0.78, 1.14) |
| 6-10 years | 0.84 | (0.24, 2.96) | 0.64 | (0.39, 1.07) | 0.99 | (0.77, 1.26) | 0.95 | (0.79, 1.15) |
| >10 years | 0.91 | (0.35, 2.31) | 0.80 | (0.54, 1.18) | 0.93 | (0.76, 1.15) | 1.10 | (0.95, 1.28) |

^a^ For women born in Oceania and West Asia, stillbirths were extremely rare in one or more groups, leading to non-estimable RRs in Poisson/GEE models in the 20 imputed data sets. We therefore present the average RR across the converged imputations, and these estimates should be interpreted as unstable.

Maternal age

Birth year

*Preconception health*

- Smoking
- Body mass index
- Pre-existing conditions
- Social disadvantage

Duration of residence stratified by

region of birth

*Structural factors*

- Antenatal care access
- Early ultrasound

*Pregnancy/perinatal outcomes*

- Gestational diabetes
- Hypterensive disorders
- Gestation at birth
- Stillbirth
- Body mass index
- Pre-existing conditions
- Social disadvantage

Unmeasured confounding

**Figure S1.** Directed acyclic graph (DAG) showing the association between duration of residence stratified by region of birth and the three groups of outcomes: preconception health, structural factors, and pregnancy/perinatal outcomes.
